# Fecal microbiota transplantation influences microbiota without connection to symptom relief in irritable bowel syndrome patients

**DOI:** 10.1038/s41522-024-00549-x

**Published:** 2024-08-28

**Authors:** Anna K. Hartikainen, Jonna Jalanka, Perttu Lahtinen, Alise J. Ponsero, Tuomas Mertsalmi, Laura Finnegan, Fiona Crispie, Paul D. Cotter, Perttu Arkkila, Reetta Satokari

**Affiliations:** 1 Human Microbiome Research Program, Faculty of Medicine, University of Helsinki, Helsinki, Finland; 2 Faculty of Medicine, University of Helsinki, Helsinki, Finland; 3 Department of Gastroenterology, Päijät-Häme Central Hospital, Lahti, Finland; 4 BIO5 Institute and Department of Biosystems Engineering, University of Arizona, Tucson, AZ USA; 5 Department of Neurology, Helsinki University Hospital HUS, Helsinki, Finland; 6 Department of Clinical Neurosciences, University of Helsinki, HUS, PO Box 800, FI-00029 Helsinki, Finland; 7 Teagasc Food Research Centre, Moorepark, Fermoy, Cork Ireland; 8 APC Microbiome, Ireland, Cork Ireland; 9 Department of Gastroenterology, Helsinki University Hospital, Helsinki, Finland

**Keywords:** Microbiome, Metagenomics

## Abstract

Imbalanced microbiota may contribute to the pathophysiology of irritable bowel syndrome (IBS), thus fecal microbiota transplantation (FMT) has been suggested as a potential treatment. Previous studies on the relationship between clinical improvement and microbiota after FMT have been inconclusive. In this study, we used 16S rRNA gene amplicon and shotgun metagenomics data from a randomized, placebo controlled FMT trial on 49 IBS patients to analyze changes after FMT in microbiota composition and its functional potential, and to identify connections between microbiota and patients’ clinical outcome. As a result, we found that the successful modulation of microbiota composition and functional profiles by FMT from a healthy donor was not associated with the resolution of symptoms in IBS patients. Notably, a donor derived strain of *Prevotella copri* dominated the microbiota in those patients in the FMT group who had a low relative abundance of *P. copri* pre-FMT. The results highlight the multifactorial nature of IBS and the role of recipient’s microbiota in the colonization of donor’s strains.

## Introduction

Irritable bowel syndrome (IBS), a functional gastrointestinal disorder, impairs patients’ quality of life by causing symptoms such as recurrent abdominal pain, changes in bowel habits and bloating^1^. Patients are classified by the predominant symptom into four subtypes: IBS with constipation (IBS-C), diarrhea (IBS-D), mixed bowel habits (IBS-M), or unclassified (IBS-U). IBS has approximately 4% prevalence worldwide^2^, although prevalence varies based on geographic region and diagnostic criteria^1,3^. The etiology and pathophysiology of the syndrome are still under investigation. Existing research recognizes that a proportion of patients have changes in their intestinal microbiota composition and function^4^. Some patients develop the syndrome after gastrointestinal (GI) infection further hinting towards association with an imbalanced microbiota^5,6^. However, the study of the gut microbiota composition studied by 16S rRNA gene sequencing has not yielded a comprehensive understanding of the role of the gut microbiota in IBS. In this context, the investigation of its functional potential and a more detailed description of the gut microbiota composition at species and strain level are critical. Furthermore, the gut microenvironment seems to play a role in IBS pathophysiology, as alterations in gut permeability, immune activity, and function of enterochromaffin cells have been linked with IBS^7^. Mucosal microbiota composition have been reported being different between IBS patients and healthy controls^8,9^, suggesting that altered microenvironment in the IBS gut may also affect the microbes residing on the mucosa and vice versa.

Currently, treatment options of IBS include, for example, non-absorbable antibiotics or probiotics or changes in diet^4^. Especially, reducing the level of fermentable oligosaccharides, disaccharides, monosaccharides and polyols (FODMAP diet) may help relieve symptoms^10^. Despite this, not all patients benefit from the current treatment options, and new treatment strategies for IBS are highly needed.

Recently, fecal microbiota transplantation (FMT) has been studied in diseases other than its recommended indication, recurrent *Clostridioides difficile* infection (rCDI). FMT has also been studied as a treatment for IBS patients with inconsistent results^11–20^. Previous studies on IBS have reported shifts in fecal microbiota composition after FMT^11,13,15,16,21–23^. However, there are contradictory findings about the association between specific bacterial taxa and IBS symptoms^21–24^. In addition, most of the IBS-FMT studies have been conducted using 16S rRNA amplicon based sequencing methods, limiting them to genus-level analysis. Only a small number of studies thus far have reported results on the functional potential of the gut microbiota or microbial metabolites in IBS-FMT trials.

Previously, we conducted a randomized, placebo-controlled clinical trial on 49 IBS patients who received a single FMT from a universal healthy donor (FMT group) or an autologous transplant as the placebo via colonoscopy (placebo group)^17^. Patients in the FMT group showed transient symptom relief at 3-month time point as compared to the baseline. In addition, FMT group patients who were classified as responders (50-point reduction in the IBS symptom severity score as compared to the baseline), showed an improvement of quality of life and reduction of mental health symptoms after the treatment. In the previous study, we also showed that bacterial richness increased in the FMT group, and that patients in the FMT group had a shift in their microbiota composition^17^. On this basis, the aim of this study was to analyze the microbiota composition in more detail up to species- and even strain-level, and whether it could be associated with symptom relief. We studied baseline fecal and colonic mucosal microbiota and engraftment of donor microbiota within the one-year follow-up. Furthermore, we aimed to address changes in functional potential of microbiota to better characterize the effect of FMT in IBS and to explore the complex interaction between the treatment-induced microbial changes and clinical outcomes.

## Results

### Fecal microbiota in the IBS patients at baseline was enriched with *Bacteroidaceae*

**Fig. 1:**
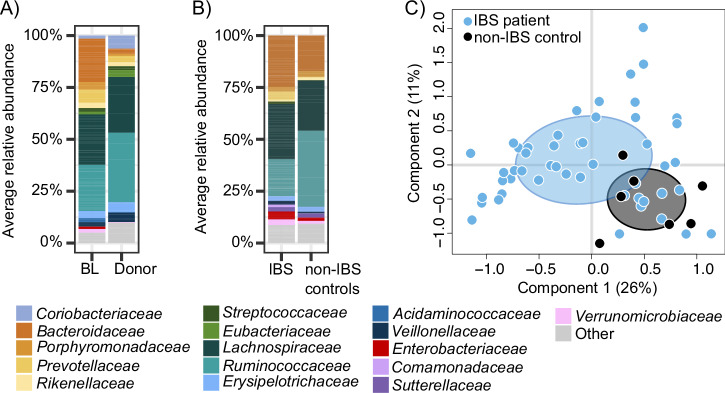
Baseline fecal and mucosal microbiota composition of the IBS patients, the donor, and the non-IBS controls. **A** Family-level average relative abundance of fecal microbiota as assessed by 16S rRNA gene amplicon sequencing showing families with >1% abundance and >20% prevalence in the IBS patients (*n* = 49) at baseline (BL) and in the donor (*n* = 1). **B** Family-level average relative abundance of mucosal microbiota as assessed by 16S rRNA gene amplicon sequencing showing families with >1% abundance and >20% prevalence in the IBS patients (IBS, *n* = 49) and the non-IBS controls (*n* = 7). **C** PCoA plot of genus-level mucosal microbiota composition as assessed by 16S rRNA gene amplicon sequencing. Measured with Bray-Curtis dissimilarity index. IBS patients grouped separately from the controls, however also dispersions of the groups differed significantly (*p* = 0.003, PERMANOVA with 999 permutations, *p* = 0.0003, betadisper).

We first profiled the fecal microbiota composition of IBS patients and a healthy donor at baseline using 16S rRNA gene amplicon sequencing. We observed that IBS patients had a high relative abundance the family *Bacteroidaceae* (Fig. 1A). More specifically, taxa from the *Bacteroidaceae* family accounted for 21.32% (±9.78) average relative abundance in IBS patients’ fecal microbiota at baseline. On the other hand, the healthy donor had a low relative abundance of family *Bacteroidaceae* (2.42% on average ± 2.66) (Fig. 1A). The genus-level fecal microbiota composition at baseline as assessed by 16S rRNA gene sequencing did not differ in PCoA between the IBS patients who were responders or non-responders at the time point of the primary outcome (12 weeks) (*p* = 0.779 for all patients, PERMANOVA with 999 permutations, Supplementary Fig. 1A). We observed the same result within patients in the FMT group (*p* = 0.833 for the FMT group, PERMANOVA with 999 permutations, Supplementary Fig. 1B). Patients’ fecal microbiota compositions were significantly more similar to other IBS patients at baseline than to the donor’s fecal composition (Bray-Curtis dissimilarity, *p* < 0.001, Wilcoxon signed rank test, Supplementary Fig. 2).

The three most dominant species as assessed by shotgun metagenomics in the IBS patients’ fecal microbiota at baseline were *Prevotella copri* (5.56% ± 13.43), *Phocaeicola dorei* (4.71% ± 6.53), and *Agathobacter rectalis* (4.15% ± 4.11) (Supplementary Fig. 3A). Notably, *Prevotella copri* had a high relative abundance in four patients at baseline (average relative abundance 37.882% ± 11.0) (Supplementary Fig. 3A). For the donor, *Gemmiger qucibialis* (10.12% ± 2.52), *Agathobacter rectalis* (5.03% ± 2.07), and *Ruminococcus E bromii B* (2.94% ± 2.62) were the three most abundant species of the fecal microbiota (Supplementary Fig. 3B).

### Mucosal microbiota composition did not differ between IBS patients and non-IBS controls

Next, we compared the baseline mucosal microbiota from the descending colon using 16S rRNA gene sequencing for the IBS patients and seven non-IBS controls (Fig. 1B). Using PERMANOVA testing at the genus level, the IBS patient mucosal microbiota was found to be significantly distinct to the non-IBS controls (Fig. 1C, *p* = 0.003, PERMANOVA with 999 permutations, *p* = 0.0003, betadisper). However, the beta-dispersion was also found to be significantly distinct between the two groups, suggesting a difference in dispersion but not in composition (Fig. 1C). This was confirmed by the absence of any taxa found to have a significantly differential abundance at the family or genus level between the two groups (Linear model on family and genus level annotations, *q* > 0.05). This result may be explained by the low sample size (49 IBS patients and 7 non-IBS controls). Additionally, we did not observe significant differences in the mucosal microbiota composition at baseline between all IBS patients or patients in the FMT group who were responders or non-responders at the time point of the primary outcome (12 weeks) (*p* = 0.373 for all patients and *p* = 0.795 for the FMT group, PERMANOVA with 999 permutations, Supplementary Fig. 4A, B).

### FMT from a healthy donor induced long-term changes in the microbiota profiles

We previously showed that 55% of the patients in FMT group had a relief in their symptoms at 12 weeks after FMT as compared to the baseline, i.e., were responders^17^. However, the relief in symptoms was transient despite a long-term shift in their microbiota composition based on 16S rRNA amplicon profiling^17^. Here, we further explored the taxa that contribute to the long-term change in the fecal microbiota profile in the FMT group.

**Fig. 2:**
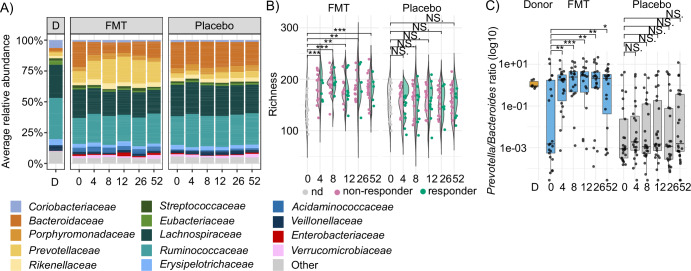
FMT induced compositional changes in the fecal microbiota of IBS patients. **A** Family-level relative abundance as assessed by 16S rRNA gene amplicon sequencing showing selected families with >1% abundance and >20% prevalence. Especially, abundance of the family *Prevotellaceae* increased in the FMT group. **B** Bacterial richness as assessed by 16S rRNA gene amplicon sequencing in the FMT (*n* = 23) and placebo (*n* = 26) groups. Violin plot shows the distribution of the data and dots represent individual samples. Richness increased significantly (Wilcoxon signed rank test, *p* < 0.01) in the FMT group as compared to the baseline but there was no significant difference between responders and non-responders (classified based on at least 50-point reduction as compared to the baseline). No increase in richness was detected in the placebo group, either in responders or non-responders. ** = *p* < 0.01, *** = *p* < 0.001, NS. = not significant. **C** *Prevotella/Bacteroides* ratio (P/B ratio) as assessed by 16S rRNA gene amplicon sequencing. Boxes show the interquartile range (IQR) of the first and third quartiles and internal line represents the median. Whiskers of the boxplot show first quartile −1.5 times IQR and third quartile +1.5 times IQR. Dots show all the individual samples. Patients in the FMT group (*n* = 23) had a significant increase in P/B ratio post-FMT compared to baseline Wilcoxon signed-rank test, * = *p* < 0.05, ** = *p* < 0.01, *** = *p* < 0.001. No significant difference was observed in the placebo group (*n* = 26) (Wilcoxon signed-rank test, NS = not significant).

At family level as assessed by 16S rRNA gene sequencing, the most noticeable changes in the fecal microbiota composition in the FMT group compared to their baseline sample were a significant increase of *Prevotellaceae* (Linear model on family level annotations, *q* > 0.05) and a significant decrease of *Bacteroidaceae* in the FMT group as compared to the baseline (Fig. 2A, Supplementary Table 2). Nevertheless, we did not observe significant differences in microbiota composition between responder and non-responder individuals at any sampling time points in the FMT group (Linear model on family level annotations, *q* > 0.05). Furthermore, the relative abundance of the genus *Prevotella* as assessed by 16S rRNA gene sequencing did not differ at baseline nor at the 12-week time point between patients who were responders or non-responders at the time point of the primary outcome (12 weeks) in the complete cohort or only within the FMT group (Wilcoxon signed rank test, Supplementary Fig. 5A–D). We observed only one significantly different family level taxa in the placebo group at 4 week-time point when compared to baseline (Supplementary Table 2). Thus, bowel lavage and autologous transplantation together resulted in a minor change in microbiota composition.

The bacterial richness increased significantly in the FMT group’s fecal microbiota compared to baseline (Wilcoxon signed rank test, *p* < 0.01), while there were no differences for the placebo group (Fig. 2B). However, no statistically significant differences between responders and non-responders were found within either group (Wilcoxon signed rank test).

Next, we assessed by 16S rRNA gene sequencing the *Prevotella*/*Bacteroides* ratio (P/B ratio), which has shown potential to differentiate individuals into different microbial enterotypes^25^. The P/B ratio of 0.01 was used as a threshold for the high/low ratio, as it was determined in the previous publications^26,27^. All samples from the donor showed a high P/B ratio (Fig. 2C). In the FMT group, the P/B ratio increased significantly, and 11 out of 12 patients transferred from the low P/B ratio category at baseline into the high P/B ratio category at the 4-week time point (Fig. 2C, Wilcoxon signed-rank test, *p* < 0.05). Whereas in the placebo group the P/B ratio did not increase significantly, and only two out of 18 patients in the low P/B ratio category at baseline changed into the high P/B ratio category at the 4-week time point (Fig. 2C, Wilcoxon signed-rank test). However, the clinical outcome was not associated to the P/B ratio, as there was no significant difference between the responders’ and non-responders’ P/B ratio at any time point in patients in the FMT group or the placebo group (Wilcoxon signed-rank test, Supplementary Fig. 6A–D).

Interestingly, we found that at 12-week time point, patients who responded to the treatment were less similar to the donor as compared to the patients who did not respond (measured with Bray-Curtis dissimilarity index and Jaccard index, *p* < 0.001 and *p* < 0.01, respectively, Wilcoxon signed rank test, Supplementary Fig. 7A, B). Moreover, we did not find evidence on the significant associations of the fecal family or genus-level composition as assessed with 16S rRNA gene sequencing with any of the measured clinical parameter (IBS-SSS, delta IBS-SSS, IBS-QOL, D15, BDI or BAI). Taken together, microbiota engraftment from the donor did not improve the symptoms, and the changes in the microbiota profiles could not be associated with the IBS symptoms or other clinical parameters.

### Baseline fecal microbiota determined the engraftment of donor *Prevotella* strains

In addition to 16S rRNA gene sequencing, we used shotgun metagenomics to assess the species-level composition and the functional potential of the microbiota. For this analysis we selected a subset of samples (133 fecal samples from 30 patients and the donor) based on the availability of the longitudinal samples and complete clinical metadata.

**Fig. 3:**
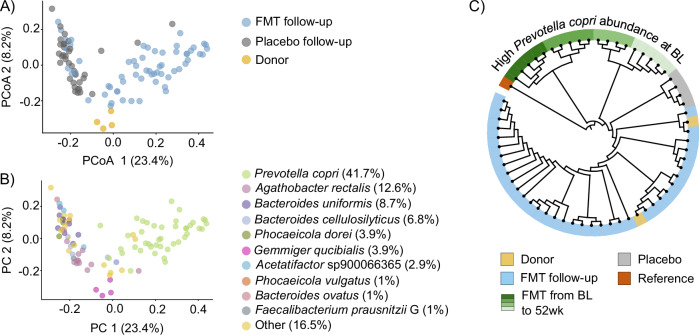
The most dominant species in each patient and the donor and *Prevotella copri* strain clustering. **A** Species-level PCoA plot as assessed by metagenomics with Bray-Curtis dissimilarity index showing separation of the FMT and placebo groups after the treatment (*p* = 0.01, PERMANOVA with 999 permutations, *p* = 0.02, betadisper). **B** The most dominant species after the treatment. *Prevotella copri* dominated the microbiota of patients in the FMT group. **C** Phylogenetic tree of the *Prevotella copri* clade A as assessed by metagenomics and StrainPhlAn. All samples grouping (marked with light blue color) with the donor samples (marked with light yellow color) were from patients in the FMT group post-FMT. Four patients from the FMT group (marked with four different shades of green) clustering with intra-individual samples had a high initial relative abundance of *P. copri*. Clade markers were found from one placebo patient (marked with gray color) and *Prevotella copri* DSM 18205 was used as a reference genome (marked with brown color).

The fecal microbiota composition of FMT and placebo patients were significantly distinct after treatment, but the difference in dispersion of the two groups was also significant (Fig. 3A, *p* = 0.001, PERMANOVA with 999 permutations, *p* = 0.02, betadisper). At the species level as assessed by metagenomics, we found that FMT from the donor changed the fecal microbiota composition profoundly. Notably, *Prevotella copri* became the dominant species in almost all patients in the FMT group throughout the follow-up period (Fig. 3B). Moreover, several other species, including *Alistipes* sp 000434235, *Coprococcus eutactus* A, and *Alistipes putredinis* were enriched in the FMT group at the follow-up time points as compared to the baseline (Linear model on species level annotations, *q* > 0.05, Supplementary Table 3).

We next investigated origin of the abundant *P. copri* in the FMT group and assessed if it was derived from the donor. Strain-level annotation was performed using the StrainPhlAn, which was able to detect markers for the highly abundant *Prevotella copri* clade A in the samples. Most of the strains were grouped together with two donor strains (Fig. 3C). Interestingly, these strains were all from patients in the FMT group post-FMT, and these patients had a low *P. copri* abundance at baseline (1.018% ± 1.91, Supplementary Fig. 2A). On the contrary, four patients with intra-individual strains, had a high relative abundance of *P. copri* at baseline (37.882% ± 11.0, Supplementary Fig. 2A). In the placebo group, clade markers were found in one patient only.

Overall, patients’ microbiotas in the FMT group were enriched with anaerobic taxa, such as *Prevotella, Faecalibacterium*, and *Alistipes*. In particular, *Prevotella copri* was enriched in patients belonging to the FMT group with a low initial *P. copri* abundance, suggesting that the strain from the donor was effective in colonizing patients and was the dominating strain post-FMT. However, a high *P. copri* abundance at baseline seemed to effectively prevent engraftment of the donor strain. Placebo-treated patients appeared to be mostly unaffected by autologous transplantation in terms of microbiota composition as assessed by metagenomics. We could not link clinical outcomes and compositional microbiota changes assessed by metagenomics in the FMT or placebo groups.

#### FMT induced long-term changes in the functional potential of the gut microbiota

**Fig. 4:**
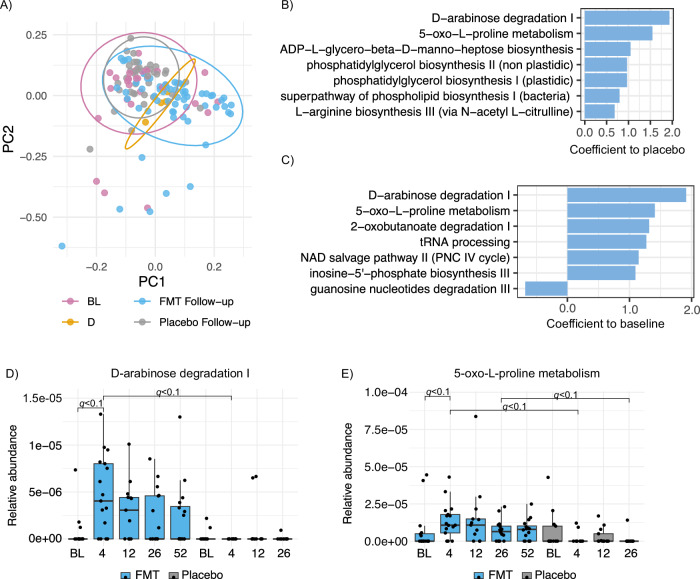
Functional potential of the luminal microbiota as assessed by metagenomic sequencing. **A** PCoA plot of the functional pathways of the donor, all patients at baseline and FMT and placebo groups at follow-up time points. Measured with Bray-Curtis dissimilarity index. FMT group was significantly different as compared to the placebo group during the follow-up (*p* = 0.001, PERMANOVA with 999 permutations, *p* = 0.30, betadisper). Moreover, the FMT follow-up samples were significantly different from the baseline samples (*p* = 0.001, PERMANOVA with 999 permutations, *p* = 0.52, betadisper). **B** Bar plot showing pathways with significant difference in the abundance between the FMT and placebo groups at 4-week time point (Linear model on functional pathways, *q* < 0.1). **C** Bar plot showing pathways with significant difference in the abundance within the FMT group between 4-week time point and baseline (Linear model on functional pathways, *q* < 0.1). **D** Relative abundance of D-arabinose degradation I pathway in the FMT and placebo groups at baseline (BL) and follow-up time points. Boxes show the interquartile range (IQR) of the first and third quartiles and internal line represents the median. Whiskers of the boxplot show first quartile −1.5 times IQR and third quartile +1.5 times IQR. Dots show all the individual samples. **E** Relative abundance of 5-oxo-L-proline metabolism pathway in the FMT and placebo groups at baseline (BL) and follow-up time points. Boxes show the interquartile range (IQR) of the first and third quartiles and internal line represents the median. Whiskers of the boxplot show first quartile −1.5 times IQR and third quartile +1.5 times IQR. Dots show all the individual samples.

We identified post-FMT changes in fecal microbiota composition and aimed to assess if this compositional change would affect functional potential of the microbial community in FMT patients. At baseline, we did not find significantly different functional pathways between the FMT and placebo groups (Linear model on functional pathways, Supplementary Table 4). During the follow-up, FMT and placebo groups differed in PCoA (*p* = 0.001, PERMANOVA with 999 permutations, *p* = 0.30, betadisper, Fig. 4A). Altogether 7, 4, and 17 functional pathways were significantly different between FMT and placebo groups at 4wk, 12wk, and 26wk, respectively (Linear model on functional pathways, *q* < 0.1, Fig. 4B, Supplementary Table 4). Of these significantly different pathways, 19 were increased in the FMT group, including several amino acid biosynthesis pathways.

Within the FMT group, the follow-up samples were significantly different from the baseline samples (*p* = 0.001, PERMANOVA with 999 permutations, *p* = 0.52, betadisper, Fig. 4A). We found 7, 38 and 1 pathways that were significantly different between baseline and follow-up time points within the FMT group at 4wk, 26wk and 52wk, respectively (Linear model on functional pathways, *q* < 0.1, Fig. 4C, Supplementary Table 5). Of these, 19 pathways were increased in the microbiota after FMT. There was a trend towards an increase in D-arabinose degradation and 5-oxo-L-proline metabolism pathways for the whole period, however significant differences were detected at 4wk for D-arabinose degradation pathway and at 4wk and 26wk for 5-oxo-proline metabolism pathway (Fig. 4D, E). Only two pathways were significantly different within the placebo group between 26 wk and baseline (Linear model on functional pathways, *q* < 0.1, Supplementary Table 6). In addition, we compared the functional potential between responders and non-responders but did not find significant differences between these groups (Linear model on functional pathways).

**Fig. 5:**
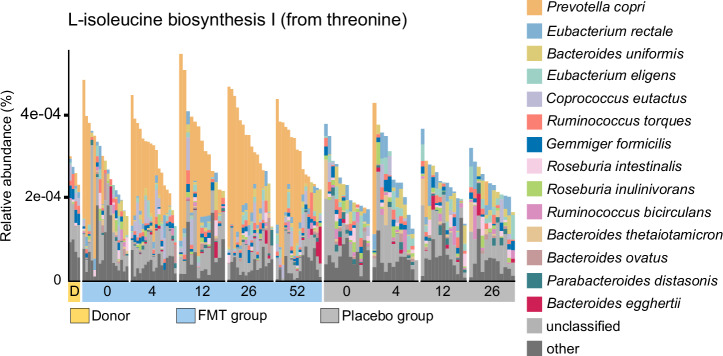
L-isoleucine biosynthesis I (from threonine) pathway in the microbiota of the donor and IBS patients. The pathway was differentially abundant between FMT and placebo groups at 26wk, and within the FMT group between 26wk and baseline (Linear model on functional pathways, *q* < 0.1, Supplementary Tables 4, 5) illustrating a high contribution of *P. copri*.

We identified eight functional pathways in which *P. copri* contributed strongly in the FMT group at 12 or 26-week time point (Supplementary Tables 4, 5). These included, the superpathway of branched chain amino acid (BCAA) biosynthesis as well as L-methionine biosynthesis IV and L-isoleucine biosynthesis I (from threonine) pathways, which were increased post FMT, and for which *P. copri* was a major contributor (Fig. 5, Supplementary Tables 4, 5).

Lastly, we aimed to associate metabolic pathways to clinical data separately in both study groups. The delta value was used in the analysis, which is the change in the value of the clinical measurements at the follow-up time points compared to the baseline. As a result, we found that metabolic pathways were not associated with the change in symptom severity (IBS-SSS) (Linear model on functional pathways, Supplementary Table 7, Supplementary Fig. 8). Within the FMT group, we found 4 and 1 pathways that were associated with the change in BAI and BDI, respectively (Linear model on functional pathways, *q* < 0.1, Supplementary Table 7, Supplementary Fig. 8A–E). One pathway, the superpathway of UPD-glucose-derived O-antigen building blocks biosynthesis, was significantly associated with both delta BAI and delta BDI. Moreover, O-antigen building blocks biosynthesis (*E. coli*) was associated with delta BAI. One pathway (dTDP-beta-D-fucofuranose biosynthesis) was significantly associated with the change in IBS-QOL within FMT group (Supplementary Table 7, Supplementary Fig. 8F).

Taken together, FMT induced drastic changes in the functional potential of the patient’s microbiota, and we observed a major, long-term shift in the metagenomic profiles in the FMT group, whereas the placebo group profiles had only minor changes. The most influenced pathways were related to biosynthesis of amino acids, especially BCAAs. The highly enriched *P. copri* contributed to several pathways post-FMT. However, functional potential did not differ between responders and non-responders. Thus, clinical outcome was not linked with functional potential of the microbiota but there were hints towards association of other clinical parameters with metabolic pathways.

## Discussion

In this study, we report the results from detailed microbiota profiling, using both 16S rRNA gene as well as shotgun metagenomics sequencing, from a randomized, placebo-controlled IBS-FMT clinical trial with 49 patients^17^. We did not find significant differences in the mucosal microbiota between IBS patients and non-IBS controls. Furthermore, we showed that a FMT from a healthy donor induced long-term changes, lasting up to one-year, in both fecal microbiota composition and its functional capacity after FMT, as well as the persistence of the donor’s strain in the recipient microbiota. Despite this, there were no significant differences in the fecal microbiota composition or functional potential between patients who responded or those who did not respond to the treatment.

There is an ongoing discussion whether a successful engraftment of the donors’ microbiota and a positive clinical outcome are associated in general and in IBS. Two recent studies utilizing metagenomics data from several different patient populations including IBS came to contradictory conclusions on this matter. On one hand, the first study showed no associations between donor strain colonization and the clinical success in general^28^. On the other hand, the second showed an association between engraftment of the donor microbiota and a positive clinical outcome for several diseases^29^. Previous studies of FMT on IBS patients specifically evaluating association of fecal microbiota composition and/or its functional potential with IBS symptoms and microbial compositional changes after FMT have reported inconsistent results. One study reported, that patients who responded to the FMT treatment showed a trend in their microbiota towards the donor microbiota composition, although the changes in microbiota functional potential could not be associated significantly with clinical success^21^. Another study found association between nine taxa and IBS-SSS, however they used other method than the standard 16S rRNA sequencing^22^. Furthermore, a recent FMT study on IBS-D patients found that while FMT did not significantly improve the patients’ IBS-SSS, it did improve the bloating severity, which was associated with a decrease of hydrogen sulphide-producing pathway and the relative abundance of bacterial taxa contributing to it^23^. Browne and colleagues showed an increase in anaerobic taxa after FMT but did not find associations between the changes in microbiota composition measured with 16S rRNA sequencing or Chao1-richness and clinical improvement^24^. Our findings replicate those by Browne and colleagues as we did find a significant increase in the bacterial richness in the FMT group only but there was no significant differences between responders and non-responders. Interestingly, in our cohort the responders’ microbiota was less similar to the donors than the non-responders’ microbiota at 12-week time point. Hence, these results further support the hypothesis that the role of microbiota in IBS pathogenesis is a complex entity and engraftment per se cannot provide symptom relief.

One of the open questions in the field remains to be the “super-donor” phenomenon, where a FMT from specific donor shows superior performance in symptom relief^30^. This has mostly been suggested with the inflammatory bowel disease patients but may also be relevant for IBS. The successful FMT trial by El-Salhy and colleagues on IBS used a so-called “super-donor” who was screened based on health criteria as well as other factors known to influence microbiota, e.g., being vaginally born, and minimal exposure to antibiotics^16,22^. This “super-donor” was also classified as “normobiotic” and shown to have a higher abundance of 12 taxa belonging to the phylum Firmicutes. While the donor of our trial was also healthy and chosen based on the recommended criteria^31^, we did not use any additional selection criteria such as the birth mode. Regardless, our donor fulfilled six out of 11 criteria defining the “super-donor”^32^. The donor’s diet is one of the factors that has been hypothesized to be particularly relevant in the “super-donor” status^30^. However, more research is critical to determine which specific diet would be beneficial. Furthermore, it is still unclear whether a single “super-donor” would suit all patients or should FMT be considered more as a personalized medicine. Although we showed a successful transfer of the donor microbiota, this could not be associated to changes in IBS symptoms. Furthermore, we found the that resident microbiota of the patients affects the colonization by donor derived strains. These results suggest that it matters which donor strains colonize the recipient and also how they function in the recipient’s ecosystem.

A previous study reported that the relative abundance of *Prevotella* was increased in the responders before FMT treatment^14^. Here we also detected a high relative abundance of *Prevotella* at baseline in a some patients, however we could not find a significant difference between the responders and non-responders. Indeed, the high abundance of *Prevotella copri* post-FMT in the current study was not linked to clinical success. An earlier study found that *Prevotella* dominated enterotype was associated with a lower IBS severity^33^. Although in the current study, we did observe a transformation from *Bacteroides* dominated enterotype into the *Prevotella* dominated enterotype for most FMT recipients, we could not link this change to any of the measured clinical parameters. One possible explanation for this might be the lack of patients presenting a severe form of IBS as well as the small sample size of our cohort.

The genus *Prevotella* has raised interest due to its dual role in GI health and disease. The most prevalent member of this group is *Prevotella copri* which has been shown to possess at least four phylogenetic clades with high genetic diversity, allowing it to thrive in different intestinal ecosystems^34^. While this bacterium has been connected to high fiber diet^35^, there are also reports suggesting that increase of *Prevotella* in the gut microbiota is associated to IBS-D and rheumatoid arthritis^36–38^. Furthermore, members of *P. copri* clade A have been reported to be among the top-engrafting species after FMT and that *P. copri* was less likely to have donor-recipient strain coexistence over other species^28,29,39^. We found a significant increase in the relative abundance of *P. copri* post-FMT and showed that the donor strain was engrafted only when the recipients’ own *P. copri* abundance was low at baseline. The observed increase in *P. copri* relative abundance could be attributed to the strain’s ability to colonize the gut effectively rather than having specific beneficial implications in IBS. Thus, niche availability or donor-recipient compatibility are important factors to be considered in FMT. Overall, the versatile functional traits of *P. copri* and these findings may help to develop FMT for other indications, especially when considering the combination of FMT treatment, e.g., with specific diet or the implications of FMT on medication. It is still unclear whether *P. copri* is beneficial or detrimental for IBS patients. Hence, its controversial role highlights further the need for development of defined microbial consortiums to eventually replace FMT treatments. This would increase the safety profile and reproducibility of the treatment as well as make it easier to implement them as a personalized medicine.

We assessed differences in the functional potential of microbiota and found differences within the FMT group as well as between the study groups. As expected, *P. copri* contributed to several pathways increased after FMT. In particular, we showed that *P. copri* contributed to pathways related to BCAA biosynthesis. Notably, a previous study has found that both lifestyle, and eating vegan, vegetarian, or an omnivore diet correlates with the selection of *P. copri* strains, which may be reflected to the genetic capacity and host-microbe associations of the strains^35^. The omnivore diet was particularly associated with higher prevalence of genes linked to BCAA biosynthesis. Moreover, another study reported high levels of BCAA in the plasma of individuals with higher levels of *P. copri*, and the bacterium was associated to insulin resistance^40^. Here, we only showed the functional potential for BCAA biosynthesis increased after FMT, but could not assess whether the host BCAA levels were changed. We also associated metabolic pathways with clinical parameters and found that there were some associations with clinical factors other than IBS symptom severity score. Especially, two O-antigen building blocks biosynthesis pathways were positively associated with the change in BAI and/or BDI. Gram-negative bacteria have lipopolysaccharides in their outer membrane, and O-antigens are part of this structure^41^. Thus, a higher capacity of O-antigen biosynthesis in the microbiota may negatively affect symptoms of anxiety and depression in IBS patients. However, the clinical trial was not designed to investigate these questions, and further studies are needed to investigate the possible connection.

To date the current study is one of the most detailed descriptions of the microbiota changes introduced by FMT treatment for IBS patients. The randomized and placebo-controlled nature of the clinical trial allowed us to appropriately compare differences between the study groups. Moreover, the clinical trial had a one-year follow-up, allowing us to address also longitudinal effects. We generated high-quality metagenomic data and used multiple analysis tools to ensure reliable results, all of which allowed us to explore in detail the associations between microbiota characteristics and clinical outcomes in our IBS patients. However, we recognize that our study has also limitations, in particular the relatively low number of patients which limits the power of the statistical analysis. Additionally, we did not follow the donor’s diet which retrospectively has been hypothesized to potentially influence treatment efficacy^32^. Moreover, the used metagenomics sequencing depth did not allow for a more detailed strain tracking and analysis of genetic composition of *P. copri* or other species of interest. Lastly, adding metatranscriptomics or metabolomics would have given additional insights that could have been valuable in understanding the links between fecal microbiota change in FMT and IBS.

In this study, a single FMT via colonoscopy was shown to effectively change the recipient’s gut microbiota composition and its functional potential in the long-term, but these changes were not associated with the clinical outcomes. Future studies must consider several aspects related to donor and patient selection and compatibility, FMT protocol, such as the route of FMT, and combination with other treatments or diet, number of patients in the clinical trials, and broader use of omics techniques to unravel the link between IBS pathophysiology and microbiota.

## Methods

### Trial design and donor

**Fig. 6:**
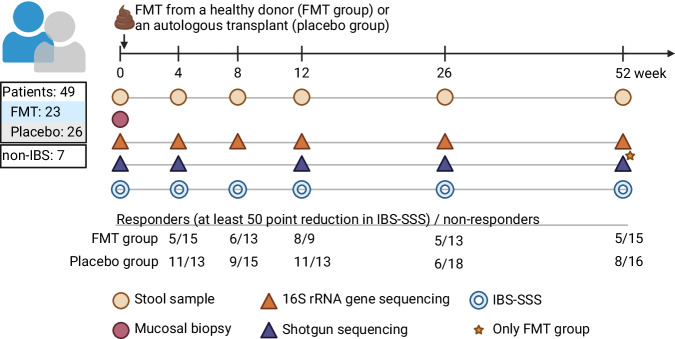
Study design. The clinical trial had 49 patients randomized into FMT (*n* = 23) and placebo (*n* = 26) groups. Stool samples were collected at six time points and mucosal biopsies at baseline. In addition, seven non-IBS controls provided a mucosal sample. Fecal samples were sequenced with 16S rRNA gene amplicon sequencing and subset of samples with shotgun metagenomics at four or five time points. At 52-week time point, only FMT group was sequenced with shotgun sequencing. Mucosal biopsies were sequenced only with 16S rRNA gene sequencing. IBS-SSS was measured at all time points. The numbers of responders and non-responders among the patients from whom IBS-SSS and 16S rRNA gene sequencing samples were available at each time point are indicated in the figure. (IBS-SSS = irritable bowel syndrome symptom severity score) Created with Biorender.com.

The detailed design of the trial has been reported previously^17^. Briefly, altogether 49 individuals with an IBS diagnosis were included into the randomized clinical trial and divided into FMT (*n* = 23) and placebo (*n* = 26) groups (Fig. 6). A FMT from a healthy donor (here referred as FMT group) or an autologous transplant (here referred as placebo group) was given via colonoscopy into the caecum. A healthy universal donor was a young male who underwent medical evaluation and laboratory tests for screening for suitable donor, as previously recommended^31^. ClinicalTrials.gov registration number of the trial is NCT03561519.

Stool samples were collected at baseline, and at 4, 8, 12, 26, and 52 weeks after FMT or placebo transfer. Mucosal biopsies from descending colon were collected at baseline. We also collected mucosal samples from subjects without IBS diagnosis (non-IBS controls, *n* = 7) for comparison purposes. The control subjects attended colonoscopy for either polyp control (*n* = 6) or because of a genetic susceptibility to colorectal cancer (*n* = 1) and were found to have normal mucosa in the endoscopy and histology.

IBS symptom score (IBS-SSS) was measured at all six time points (Fig. 6). We categorized patients as responders or non-responders as described in the original publication^17^. Briefly, patients were considered as responders if they achieved at least 50-point decrease in IBS-SSS at any time point as compared to the baseline value (Fig. 6). Furthermore, we used the responder status at the time point of the primary outcome (12 weeks) to assess whether the baseline microbiota composition would differ between responders and non-responders. Moreover, patients provided other questionnaires including two quality of life measurements (IBS-QOL, 15D), Beck’s Depression Inventory (BDI) and Beck Anxiety Inventory (BAI).

### Ethics

All patients received trial info and they gave written informed consent to participate. The clinical trial was approved by the ethical committee of Helsinki University Hospital (registration number 40/13/03/01/2015). The samples from the non-IBS controls were collected with the approval HUS 29/13/03/01/2014.

### Sample preparation

Stool DNA was extracted with a previously described method using repeated bead beating as the mechanical cell lysis step^42^. High-throughput DNA purification was made with the KingFisher™ Flex Purification System, KingFisher with 96 PCR head (Thermo Fisher Scientific, Vantaa, Finland). Mucosal DNA extraction included mechanical and chemical cell lysis^43^. Fecal DNA concentration was measured with Quant-iT™ dsDNA High-Sensitivity Assay Kit (Thermo Fisher Scientific, Eugene, Oregon, USA) and mucosal with NanoDrop™ 2000 Spectrophotometer (Thermo Fisher Scientific). All samples were delivered for sequencing (Teagasc Next Generation DNA Sequencing Facility, Cork, Ireland).

### Sequencing and sequence processing

Mucosal and fecal microbiota profiling of all time points and patients was done by MiSeq (Illumina, San Diego, California, USA) 16S rRNA gene amplicon sequencing of the hypervariable region V3-V4 (in total 279 fecal samples from 49 patients and donor, and mucosal samples from 49 patients and 7 non-IBS controls). 16S rRNA gene based microbial profiling was done with R package mare^44^ pipeline. Forward reads were quality-filtered (truncQ = 2, maxEE = 1), low-abundance reads (<0.001% for fecal data and 0.01% for mucosal data), chimeras were removed, and reads were truncated into 180 bp length. Taxonomic annotation was made with USEARCH^45^ (v11) against the RDP database (RDP training set v16). One mucosal sample with less than 10,000 reads was discarded. Average read count after processing was 41,728 (±7875) for fecal samples and 31,831 (±9722) for mucosal samples.

Moreover, we conducted a shotgun metagenomic sequencing for 133 fecal samples from 30 patients and the donor. Subset of patients were selected based on the availability of the longitudinal samples and complete clinical metadata. Nextera XT library preparation kit was used for preparing the sequencing library, and sequencing was conducted with NextSeq 550 (high output, 300 cycles). Sequences were first quality controlled with FastQC and MultiQC softwares. Next, TRIMMOMATIC^46^ (version 0.39) was used for trimming adapter sequences and sequences were quality-filtered with parameters: ‘ILLUMINACLIP:NexteraPE-PE.fa:2:30:10:2:True LEADING:3 TRAILING:3 MINLEN:50’. Then human reads were removed using Bowtie2^47,48^ (version 2.3.5.1) aligning against the human chromosomal database (GRCh38), and then using samtools^49^ (1.16.1) to filter unmapped reads and split paired-end reads into separated files. Average read count after processing was 3,748,621 (±1,497,298). For taxonomic assignment, Kraken2^50^ (version 2.1.2) and Bracken^51,52^ (version 2.7.0) were used against the HumGut^53^ database. Functional annotation was conducted with HUMAnN3^54^ (version 3.0.1).

Both 16S rRNA gene amplicon and shotgun metagenomics data are publicly available at ENA under accession number PRJEB65418.

### Statistical analysis

Statistical analyses were performed using R software^55^ (version 4.3.0) and R-studio^56^ (version 2023.06.0). Figures were created with ggplot2^57^ and miaViz^58^ R packages. Microbiota composition comparison was done by calculating Bray-Curtis dissimilarity with relative abundances or Jaccard index (using R package vegan^59^), and visualized with Principal Coordinates Analysis (PCoA) plot (with either ‘PCoA’ function in R package mare^44^ or ‘plotReduceDim’ in R package scater^60^). Permutational analysis of variance (PERMANOVA) was used to test statistical significance in the microbiota composition between selected groups. Function ‘adonis2’ in R package vegan^59^ was used with relative abundance data, Bray-Curtis dissimilarity, and 999 permutations. The homogeneity of the group dispersions was calculated with PERMDISP2 method and functions ‘anova’, and ‘betadisper’ in R package vegan^59^. The most dominant species was visualized with a modified code from ‘Orchestrating Microbiome Analysis with R and Bioconductor’^61^. Richness estimates were generated with R package vegan^59^. The statistical differences between groups or time points were measured with Wilcoxon signed rank test (for non-parametric data).

Differential abundance testing for 16S rRNA gene sequencing (for family-level data) and metagenomics data (for species-level data and functional profiles) was done with MaAsLin2 function in Maaslin2^62^ R package. We used square-root transformation and total sum scaling normalization for the non-rarefied count data. We specified ‘min_abundance’ at 0.0001 and ‘min_prevalence’ at 0.1. When we used longitudinal samples, we specified patient ID as a random effect. MaAsLin2 was also used for unstratified CPM normalized functional profiles with the default transformation and normalization and similar prevalence and abundance filtering as with the microbiota data. Function fitted a linear model, tested significance with a Wald test, and gave output as a BH FDR-corrected *p* values (*q*-value). Results with *q* value of <0.05 and <0.1 were considered as significant for the microbiota and functional profiles, respectively.

We used StrainPhlAn^63,64^ for strain characterization with the taxonomic and functional annotation in selected species with a high relative abundance. First, MetaPhlAn^63^ (version 4.0.2) was used to get taxonomical and functional annotation. Then, StrainPhlAn^64^ (version 4.0.6) was ran with default parameters, except: ‘—marker_in_n_samples 5’, ‘—sample_with_n_markers 5’ and using *Prevotella copri* clade A marker SGB1626. The genome assembly of *Prevotella copri* DSM 18205 (GCA_020735445.1, now suggested to be *Segatella copri*) was used as a reference genome. The resulting phylogenetic tree was visualized with MEGA X^65,66^ (version 10.2.6) and modified with iTol^67^ (version 6).

We aimed to connect the microbiota composition and its functional potential with the clinical outcome in multiple ways. We compared bacterial richness, microbiota similarity to donor (measured with Bray-Curtis dissimilarity index and Jaccard index), microbiota composition and its functional capacity between responders and non-responders. Moreover, we used ‘CovariateTest’ in R package mare^44^ to identify bacterial genera or families that would have associations with IBS-SSS or delta IBS-SSS (change in the IBS-SSS as compared to the baseline), BDI, BAI, 15D, or IBS-QOL scores (a detailed description of covariates can be found in Supplementary Table 1). Function first fits negative binomial model, linear models, generalized least squares models or linear mixed model to the data using read count as an offset. Mixed models were used for non-independent samples. The chosen model estimates the significance between bacterial taxa and measured score. Furthermore, we used MaAsLin2 associate changes in clinical parameters (delta IBS-SSS, delta BAI, delta BDI, delta IBS-QOL, delta 15D, description in Supplementary Table 1) with the metabolic pathways separately in both study groups.

### Supplementary information

Supplementary information

Report Summary

## Data Availability

Both 16S rRNA gene amplicon and shotgun metagenomics data are publicly available at ENA under accession number PRJEB65418. Clinical samples used for microbiota analysis are not available for other researchers due to restrictions on distribution of clinical material.

## Code availability

The code used for the bioinformatic analysis and statistics is available upon request to the corresponding author.
