# Supplementary material for: Fecal microbiota transplantation influences microbiota without connection to symptom relief in irritable bowel syndrome patients
Source: NPJ Biofilms Microbiomes. 2024 Aug 28;10:73. doi: 10.1038/s41522-024-00549-x (PMC11349920; doi:10.1038/s41522-024-00549-x)
Supplement: Supplementary file 1 — Supplementary information [file 41522_2024_549_MOESM1_ESM.pdf]

Supplementary Figures

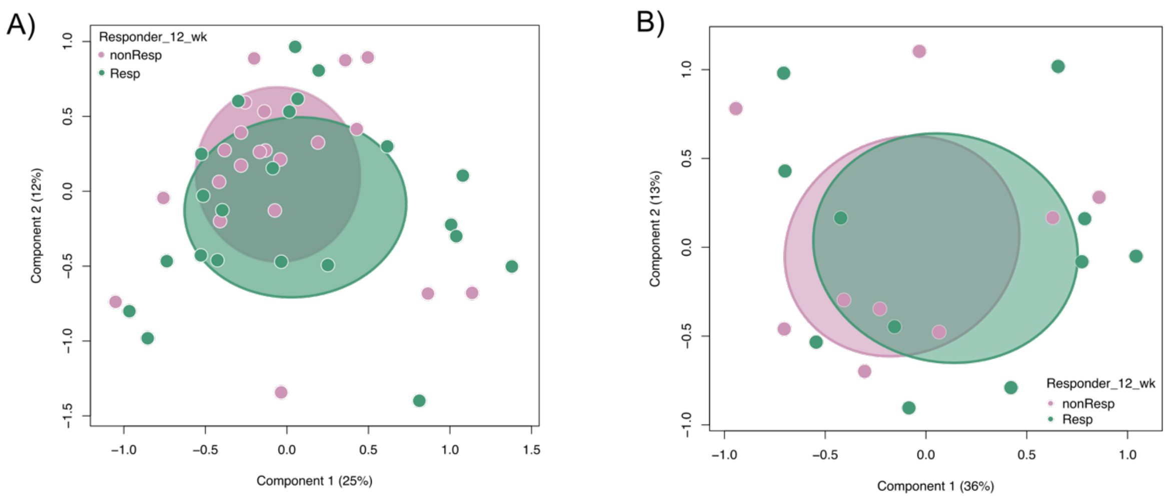

Supplementary Figure 1. PCoA plot of the genus-level luminal microbiota composition at baseline. Assessed by 16S rRNA gene amplicon sequencing showing responder status at 12 week time-point (nonResp=non-responder, Resp=responder). Measured with Bray-Curtis dissimilarity index. **(A)** All patients at baseline. No significant difference was found. (p=0.779, PERMANOVA with 999 permutations). **(B)** Only patients in FMT group at baseline. No significant difference was found. (p=0.833, PERMANOVA with 999 permutations).

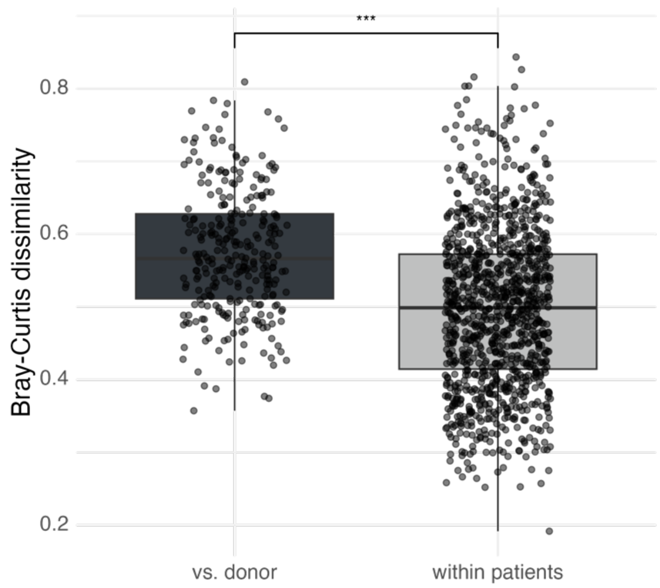

Supplementary Figure 2. Genus-level beta-diversity at baseline. (A) Bray-Curtis dissimilarity index between patient samples and donor samples (vs. donor) and within patients at baseline. Significant difference was found (***=p<0.001, Wilcoxon signed rank test). Boxes show the interquartile range (IQR) of the first and third quartiles and internal line represents the median. Whiskers of the boxplot show first quartile - 1.5 times IQR and third quartile + 1.5 times IQR. Dots show all the individual samples.

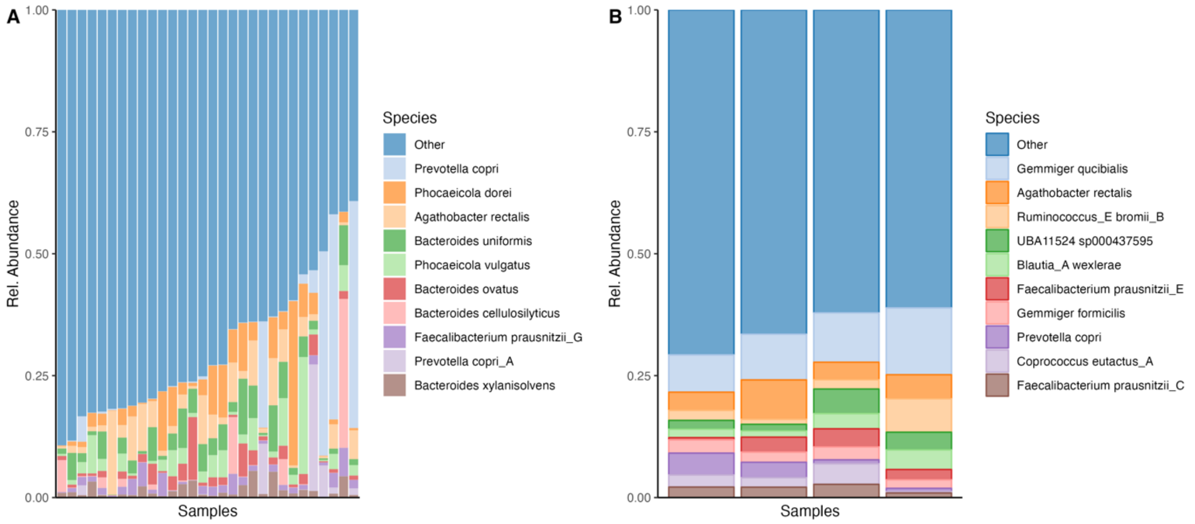

Supplementary Figure 3. Luminal species-level relative abundance in the IBS patients at baseline and in the healthy donor. (A) Species-level relative abundance of luminal microbiota as assessed by metagenomics showing 10 species with the highest relative abundance in the IBS patients at baseline. **(B)** Species-level relative abundance of luminal microbiota as assessed by metagenomics showing 10 species with the highest relative abundance in the healthy donor.

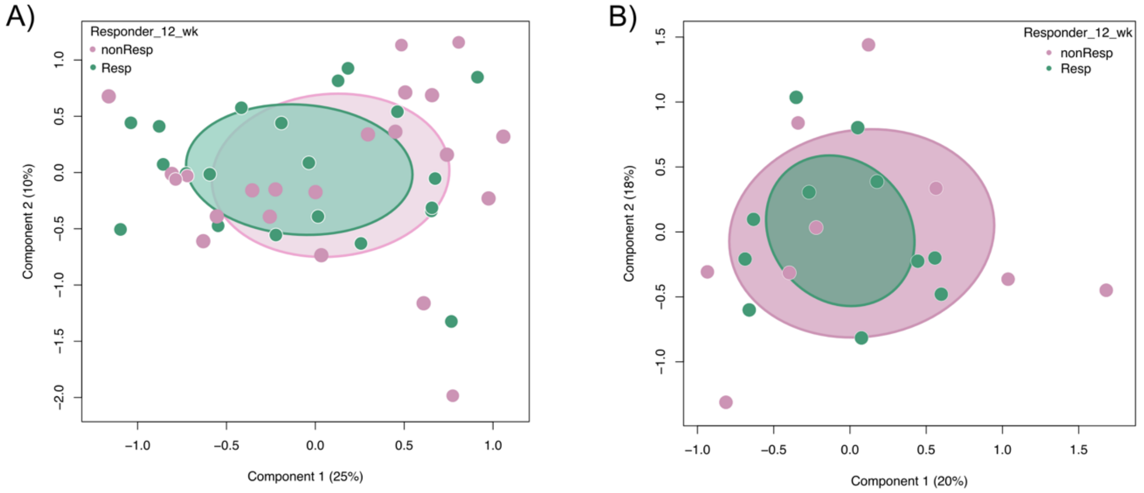

Supplementary Figure 4. PCoA plot of the genus-level mucosal microbiota composition at baseline. Assessed by 16S rRNA gene amplicon sequencing at baseline showing responder status at 12-week time-point (nonResp=non-responder, Resp=responder). Measured with Bray-Curtis dissimilarity index. **(A)** All patients. No significant difference was found. (p=0.373, PERMANOVA with 999 permutations). (B) Only patients in FMT group. (p=0.795, PERMANOVA with 999 permutations).

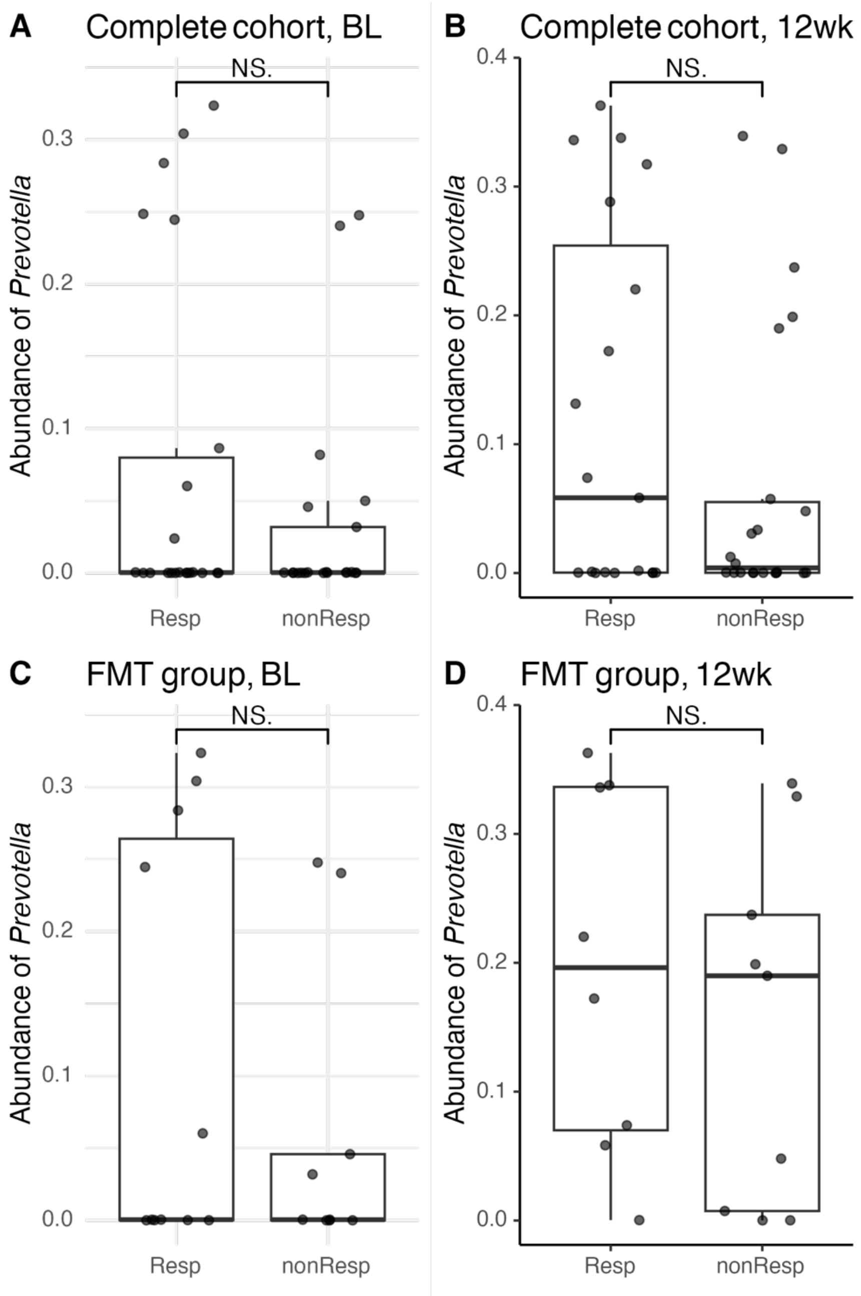

Supplementary Figure 5. Relative abundance of the luminal genus *Prevotella* and responder status. Assessed by 16S rRNA gene sequencing at baseline **(A)** and at 12wk **(B)** in the complete cohort in responders and non-responders (responder status at 12 wk time point). No significant difference was found (Wilcoxon signed rank test, NS = not significant). Assessed by 16S rRNA gene sequencing at baseline **(C)** and at 12wk **(D)** in the FMT group in responders and non-responders (responder status at 12 wk time point). No significant difference was detected (Wilcoxon signed rank test, NS = not significant). Boxes show the interquartile range (IQR) of the first and third quartiles and internal line represents the median. Whiskers of the boxplot show first quartile - 1.5 times IQR and third quartile + 1.5 times IQR. Dots show all the individual samples.

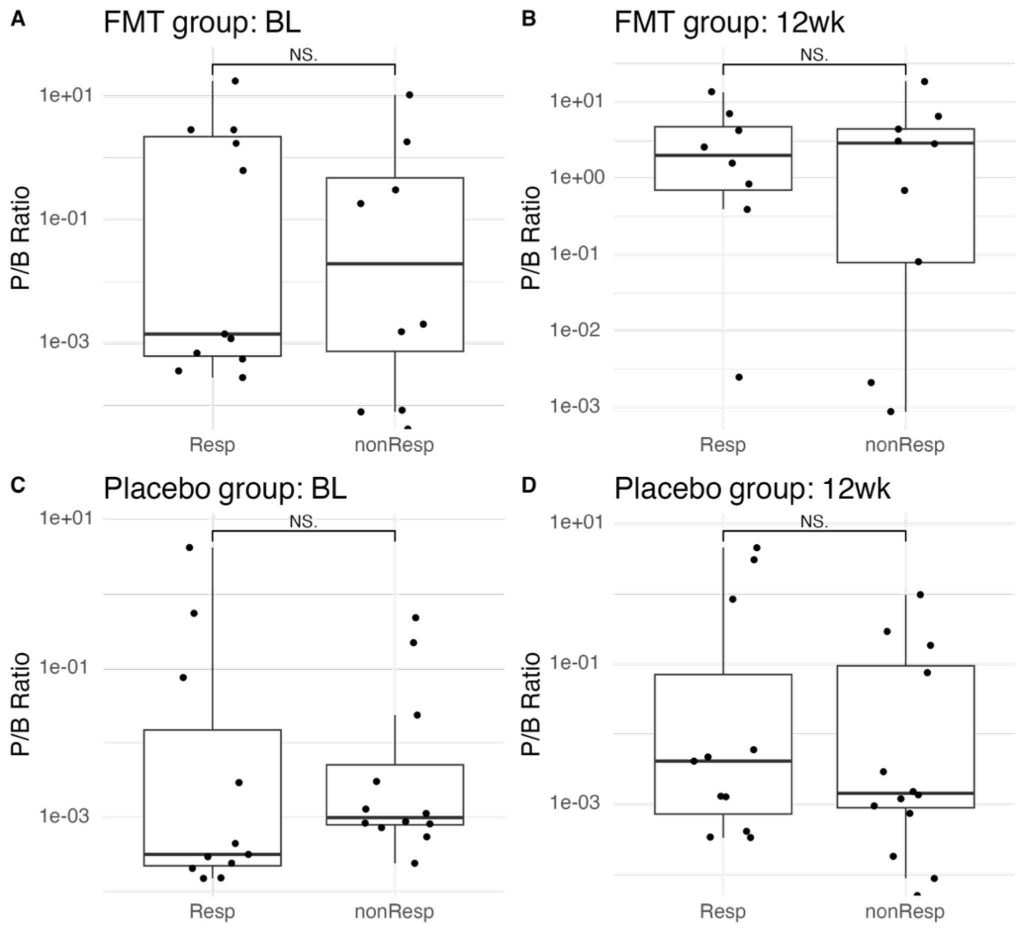

Supplementary Figure 6. Luminal *Prevotella*/*Bacteroides* ratio (P/B ratio). Assessed by 16S rRNA gene sequencing in the FMT group at baseline **(A)** and at 12 wk **(B)**, in the placebo group at BL **(C)** and at 12 wk **(D)** in responders (Resp) and non-responders (nonResp) (responder status at 12 wk time point). No significant difference was found (Wilcoxon signed rank test, NS = not significant). Boxes show the interquartile range (IQR) of the first and third quartiles and internal line represents the median. Whiskers of the boxplot show first quartile - 1.5 times IQR and third quartile + 1.5 times IQR. Dots show all the individual samples.

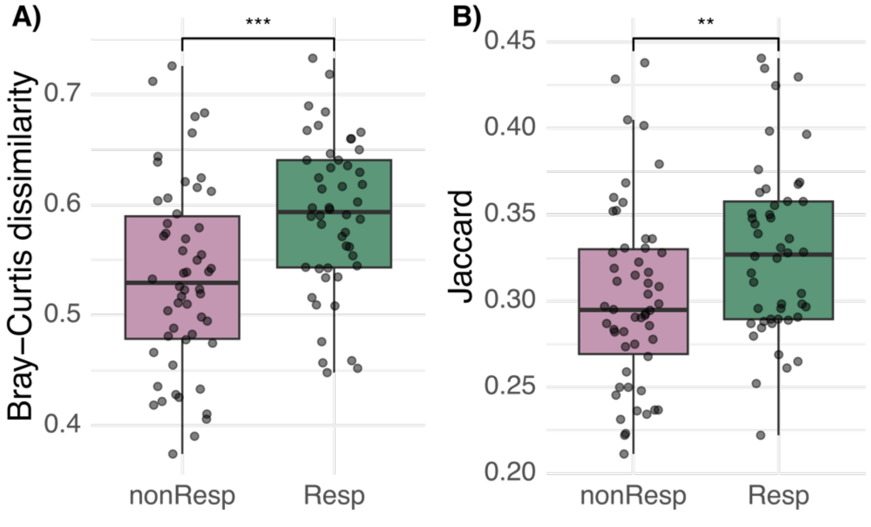

Supplementary Figure 7. Genus-level beta-diversity at baseline in responders and non-responders. (A) Bray-Curtis dissimilarity index to the donor samples at 12wk in the FMT group showing responder status (nonResp=non-responder, Resp=responder). Significant difference was found (***=p<0.001, Wilcoxon signed rank test). **(B)** Jaccard index to the donor samples at 12wk in the FMT group showing responder status

(nonResp=non-responder, Resp=responder). Significant difference was found (**=p<0.01, Wilcoxon signed rank test). Boxes show the interquartile range (IQR) of the first and third quartiles and internal line represents the median. Whiskers of the boxplot show first quartile - 1.5 times IQR and third quartile + 1.5 times IQR. Dots show all the individual samples.

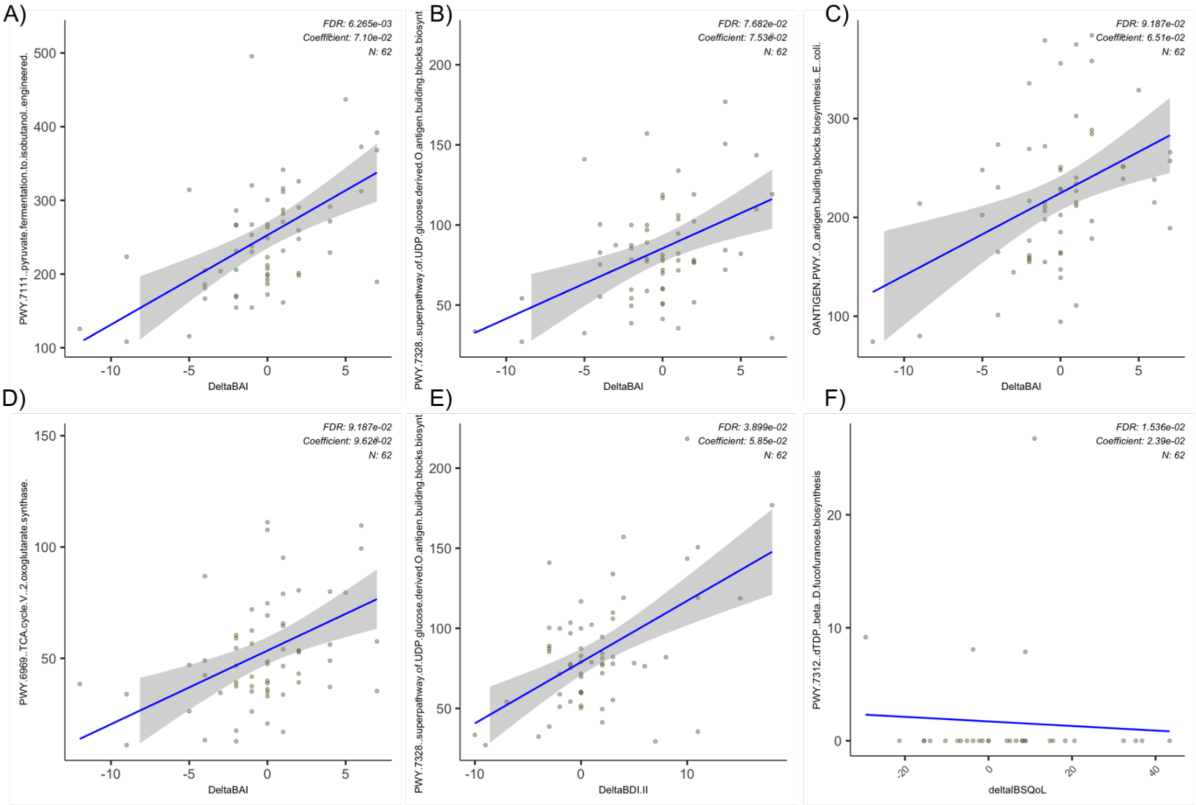

Supplementary Figure 8. Metabolic pathway associations with clinical data. (A) Association of PWY-7111: pyruvate fermentation to isobutanol (engineered) with delta BAI within FMT group in follow-up samples. **(B)** Association of PWY-7328: superpathway of UDP-glucose-derived O-antigen building blocks biosynthesis with delta BAI within FMT group in follow-up samples. **(C)** Association of OANTIGEN-PWY: O-antigen building blocks biosynthesis (*E. coli*) with delta BAI within FMT group in follow-up samples. **(D)** Association of PWY-6969: TCA cycle V (2-oxoglutarate synthase) with delta BAI within FMT group in follow-up samples. **(E)** Association of PWY-7328: superpathway of UDP-glucose-derived O-antigen building blocks biosynthesis with delta BDI within FMT group in follow-up samples. **(F)** Association of PWY-7312: dTDP-β-D-fucofuranose biosynthesis with delta IBS-QOL within FMT group in follow-up samples. Dots show all the individual samples.

Supplementary table 1. Covariate description

Variable	Description
IBS-SSS	Irritable Bowel Syndrome Symptom Severity Score
delta IBS-SSS	Irritable Bowel Syndrome Symptom Severity Score compared to the baseline
BAI	Beck Anxiety Inventory
delta BAI	Beck Anxiety Inventory compared to the baseline
BDI	Beck’s Depression Inventory
delta BDI	Beck’s Depression Inventory compared to the baseline
IBS-QOL	Irritable Bowel Syndrome Quality of Life
delta IBS-QOL	Irritable bowel syndrome Quality of Life compared to the baseline
15D	Quality of Life
delta 15D	Quality of Life compared to the baseline

Supplementary table 2. Differential abundance testing on luminal 16S rRNA gene amplicon sequencing at family-level with Maaslin2

**Baseline: between patients who will be in FMT and placebo groups, family-level**
No significant differences

**4wk: FMT vs. Placebo, family level**								
**feature**	**metadata**	**value**	**coef**	**stderr**	**N**	**N.not.0**	**pval**	**qval**
Bacteroidetes_Bacteroidia_Bacteroidales_Prevotellaceae	GroupLong	FMT	0,25777381	0,04903319	46	46	4,11E-06	0,00011293
Firmicutes_Negativicutes_Selenomonadales_Acidaminococcaceae	GroupLong	FMT	0,13150243	0,02475881	46	34	3,43E-06	0,00011293
Bacteroidetes_Bacteroidia_Bacteroidales_Bacteroidaceae	GroupLong	FMT	-0,1160468	0,03204471	46	46	0,00075391	0,01382173
Firmicutes_Clostridia_Clostridiales_Lachnospiraceae	GroupLong	FMT	-0,0590289	0,0169441	46	46	0,00113113	0,01555305
**12wk: FMT vs. Placebo, family level**								
**feature**	**metadata**	**value**	**coef**	**stderr**	**N**	**N.not.0**	**pval**	**qval**
Bacteroidetes_Bacteroidia_Bacteroidales_Bacteroidaceae	GroupLong	FMT	-0,1715383	0,0362374	44	44	2,52E-05	0,00069163
Bacteroidetes_Bacteroidia_Bacteroidales_Prevotellaceae	GroupLong	FMT	0,29392814	0,06116641	44	44	2,00E-05	0,00069163
Firmicutes_Negativicutes_Selenomonadales_Acidaminococcaceae	GroupLong	FMT	0,10069203	0,02437857	44	36	0,00016858	0,00309064
Firmicutes_Bacilli_Bacillales_Staphylococcaceae	GroupLong	FMT	0,00366571	0,00100799	44	11	0,0007486	0,01029319

**52wk: FMT vs. Placebo, family level**								
**feature**	**metadata**	**value**	**coef**	**stderr**	**N**	**N.not.0**	**pval**	**qval**
Firmicutes_Negativicutes_Selenomonadales_Acidaminococcaceae	GroupLong	FMT	0,10601833	0,02458151	47	39	8,69E-05	0,00486692
Bacteroidetes_Bacteroidia_Bacteroidales_Prevotellaceae	GroupLong	FMT	0,23261701	0,05860324	47	46	0,00025657	0,00718403
Bacteroidetes_Flavobacteriia_Flavobacteriales_Flavobacteriaceae	GroupLong	FMT	0,01001814	0,00291882	47	18	0,00129446	0,02416323
**FMT 4 wk vs. BL, family level, random effects = "PatientID"**								
**feature**	**metadata**	**value**	**coef**	**stderr**	**N**	**N.not.0**	**pval**	**qval**
Bacteroidetes_Bacteroidia_Bacteroidales_Bacteroidaceae	Timepoint	4wk	-0,151084	0,02818193	44	44	3,29E-05	0,0018084
Synergistetes_Synergistia_Synergistales_Synergistaceae	Timepoint	4wk	0,04078315	0,00862443	44	38	0,00011199	0,00307981
Bacteroidetes_Bacteroidia_Bacteroidales_Rikenellaceae	Timepoint	4wk	0,072692	0,01642593	44	43	0,0002207	0,00404613
Firmicutes_Clostridia_Clostridiales_Clostridiales_Incertae_Sedis_XI	Timepoint	4wk	0,01386834	0,00350788	44	31	0,00068291	0,00939007
Bacteroidetes_Flavobacteriia_Flavobacteriales_Flavobacteriaceae	Timepoint	4wk	0,01169746	0,00312809	44	13	0,00118108	0,01299185
Firmicutes_Bacilli_Lactobacillales_Carnobacteriaceae	Timepoint	4wk	-0,0048686	0,00142706	44	26	0,00256622	0,02352367
Bacteroidetes_Bacteroidia_Bacteroidales_Prevotellaceae	Timepoint	4wk	0,13933759	0,04411441	44	44	0,00478914	0,03762894
Firmicutes_Bacilli_Bacillales_Bacillaceae_2	Timepoint	4wk	0,0096887	0,00325666	44	21	0,007082	0,04868876

FMT 8 wk vs. BL, family level, random effects = "PatientID"

feature	metadata	value	coef	stderr	N	N.not.0	pval	qval
Bacteroidetes_Bacteroidia_Bacteroidales_Bacteroidaceae	Timepoint	8wk	-0,1716877	0,02606196	41	41	2,89E-06	0,00016168
Bacteroidetes_Bacteroidia_Bacteroidales_Prevotellaceae	Timepoint	8wk	0,20453551	0,04418416	41	41	0,00019462	0,00363295
Firmicutes_Clostridia_Clostridiales_Clostridiales_Incertae_Sedis_XI	Timepoint	8wk	0,0166556	0,0036407	41	29	0,00017244	0,00363295
Bacteroidetes_Bacteroidia_Bacteroidales_Rikenellaceae	Timepoint	8wk	0,07424076	0,01768292	41	40	0,00042795	0,00599135
Bacteroidetes_Flavobacteriia_Flavobacteriales_Flavobacteriaceae	Timepoint	8wk	0,01027899	0,00276636	41	13	0,00129559	0,01451065
Actinobacteria_Actinobacteria_Actinomycetales_Micrococcaceae	Timepoint	8wk	0,01302766	0,00390232	41	26	0,001862	0,01484287
Firmicutes_Bacilli_Bacillales_Bacillaceae_2	Timepoint	8wk	0,01182911	0,00359943	41	22	0,00215222	0,01484287
Firmicutes_Bacilli_Bacillales_Staphylococcaceae	Timepoint	8wk	0,00443722	0,00135701	41	10	0,00225313	0,01484287
Proteobacteria_Deltaproteobacteria_Syntrophobacterales_Syntrophaceae	Timepoint	8wk	0,00644118	0,0019824	41	10	0,00238546	0,01484287
Firmicutes_Clostridia_Clostridiales_Peptococcaceae_2	Timepoint	8wk	0,0099449	0,00296149	41	32	0,00319923	0,01791566
Synergistetes_Synergistia_Synergistales_Synergistaceae	Timepoint	8wk	0,04376083	0,01362241	41	35	0,00416913	0,02122466

FMT 12 wk vs. BL, family level, random effects = "PatientID"

feature	metadata	value	coef	stderr	N	N.not.0	pval	qval
Bacteroidetes_Bacteroidia_Bacteroidales_Bacteroidaceae	Timepoint	12wk	-0,1727671	0,02736595	40	40	5,74E-06	0,0003213
Bacteroidetes_Bacteroidia_Bacteroidales_Prevotellaceae	Timepoint	12wk	0,20757146	0,05374845	40	40	0,00107893	0,02630507

Firmicutes_Bacilli_Bacillales_Staphylococcaceae	Timepoint	12wk	0,00385707	0,00105583	40	10	0,0014092	0,02630507
Proteobacteria_Gammaproteobacteria_Pseudomonadales_Pseudomonadaceae	Timepoint	12wk	-0,0031958	0,00089989	40	18	0,00211259	0,02957631
Firmicutes_Bacilli_Bacillales_Bacillaceae_2	Timepoint	12wk	0,00961268	0,00310405	40	21	0,00366663	0,0410662
Bacteroidetes_Flavobacteriia_Flavobacteriales_Flavobacteriaceae	Timepoint	12wk	0,00812865	0,00269288	40	9	0,00690117	0,04830822
Firmicutes_Clostridia_Clostridiales_Clostridiales_Incertae_Sedis_XI	Timepoint	12wk	0,01131196	0,00373005	40	29	0,00660934	0,04830822
Synergistetes_Synergistia_Synergistales_Synergistaceae	Timepoint	12wk	0,03195508	0,0105543	40	34	0,00648889	0,04830822
**FMT 26 wk vs. BL, family level, random effects = "PatientID"**								
**feature**	**metadata**	**value**	**coef**	**stderr**	**N**	**N.not.0**	**pval**	**qval**
Bacteroidetes_Bacteroidia_Bacteroidales_Bacteroidaceae	Timepoint	26wk	-0,1368665	0,02730423	42	42	7,14E-05	0,00399646
Firmicutes_Clostridia_Clostridiales_Clostridiales_Incertae_Sedis_XI	Timepoint	26wk	0,01105234	0,00252351	42	29	0,00028712	0,00803942
Synergistetes_Synergistia_Synergistales_Synergistaceae	Timepoint	26wk	0,03123144	0,00771138	42	36	0,00061196	0,01142325
Bacteroidetes_Bacteroidia_Bacteroidales_Prevotellaceae	Timepoint	26wk	0,17956678	0,04995166	42	42	0,00182844	0,02047851
Bacteroidetes_Flavobacteriia_Flavobacteriales_Flavobacteriaceae	Timepoint	26wk	0,00984445	0,00271016	42	13	0,00156618	0,02047851
Firmicutes_Bacilli_Bacillales_Bacillaceae_2	Timepoint	26wk	0,00784829	0,00240896	42	18	0,00381661	0,03562167
**FMT 52 wk vs. BL, family level, random effects = "PatientID"**								
**feature**	**metadata**	**value**	**coef**	**stderr**	**N**	**N.not.0**	**pval**	**qval**

Firmicutes_Clostridia_Clostridiales_Clostridiales_Incertae_Sedis_XI	Timepoint	52wk	0,01573329	0,00245726	43	33	2,62E-06	0,00014681
Bacteroidetes_Flavobacteriia_Flavobacteriales_Flavobacteriaceae	Timepoint	52wk	0,01060069	0,00257756	43	14	0,00050108	0,01403014
Synergistetes_Synergistia_Synergistales_Synergistaceae	Timepoint	52wk	0,02799677	0,0073298	43	36	0,00101859	0,01901372
Firmicutes_Bacilli_Bacillales_Bacillaceae_2	Timepoint	52wk	0,0081088	0,00226918	43	21	0,00179711	0,02515949
Bacteroidetes_Bacteroidia_Bacteroidales_Bacteroidaceae	Timepoint	52wk	-0,1100647	0,0317558	43	43	0,00237414	0,02659042
**Placebo 4 wk vs. BL, family level, random effects = "PatientID"**								
**feature**	**metadata**	**value**	**coef**	**stderr**	**N**	**N.not.0**	**pval**	**qval**
Firmicutes_Bacilli_Lactobacillales_Carnobacteriaceae	Timepoint	4wk	-0,0049482	0,00123473	49	30	0,00052727	0,0279455

**Placebo 8wk vs. BL, family level, random effects = "PatientID"**
No significant differences
**Placebo 12wk vs. BL, family level, random effects = "PatientID"**
No significant differences
**Placebo 26wk vs. BL, family level, random effects = "PatientID"**
No significant differences

**Placebo 52wk vs. BL, family level, random effects = "PatientID"**
No significant differences

Supplementary table 3. Differential abundance testing on luminal metagenomics at species-level with Maaslin2
FMT group follow-up time points vs. baseline

feature	metadata	value	coef	stderr	N	N.not.0	pval	qval
**4wk vs. BL, random effects: "ID"**								
Alistipes.sp900548155	GroupTP	Tx4	0,00761945	0,00144171	34	33	8,67E-06	0,00136989
Faecalibacterium.prausnitzii_E	GroupTP	Tx4	0,05972206	0,01129767	34	34	8,64E-06	0,00136989
CAG.83.sp900549395	GroupTP	Tx4	0,00523546	0,00083818	34	34	1,17E-05	0,00136989
CAG.238.sp900551415	GroupTP	Tx4	0,01605387	0,00263252	34	34	1,54E-05	0,00136989
UMGS1603.sp900553265	GroupTP	Tx4	0,00658428	0,00100836	34	33	6,94E-06	0,00136989
UMGS1633.sp900553645	GroupTP	Tx4	0,01093667	0,00178728	34	32	1,48E-05	0,00136989
Holdemanella.sp003436425	GroupTP	Tx4	0,00651373	0,00125497	34	32	1,14E-05	0,00136989
Prevotella.sp000435635	GroupTP	Tx4	0,00546245	0,00092727	34	33	2,28E-05	0,00157705
Alistipes.sp000434235	GroupTP	Tx4	0,10737163	0,01805499	34	34	2,05E-05	0,00157705
Ruminococcus_C.callidus	GroupTP	Tx4	0,03429656	0,00704395	34	34	2,91E-05	0,00181138
CAG.170.sp900553545	GroupTP	Tx4	0,01584387	0,00279529	34	33	3,49E-05	0,00197907
SFFH01.sp900548125	GroupTP	Tx4	0,01062155	0,00222568	34	34	3,85E-05	0,00199756
Alistipes.shahii	GroupTP	Tx4	0,03205271	0,00588284	34	34	5,36E-05	0,00207636
Alistipes_A.sp900240235	GroupTP	Tx4	0,01386732	0,00302642	34	33	6,67E-05	0,00207636
ER4.sp900317525	GroupTP	Tx4	0,03226226	0,00604213	34	34	6,64E-05	0,00207636
ER4.sp900552015	GroupTP	Tx4	0,00755201	0,00163823	34	33	6,15E-05	0,00207636
CAG.103.sp900543625	GroupTP	Tx4	0,03050916	0,00570147	34	34	6,49E-05	0,00207636
UMGS1696.sp900554225	GroupTP	Tx4	0,01587383	0,00292146	34	29	5,52E-05	0,00207636
Olsenella_E.sp003609875	GroupTP	Tx4	0,00941478	0,0017064	34	34	4,68E-05	0,00207636
Victivallis.sp002998355	GroupTP	Tx4	0,01468147	0,00320098	34	33	6,58E-05	0,00207636
CAG.345.sp000433315	GroupTP	Tx4	0,00483356	0,00094514	34	31	0,00010404	0,0030864
Alistipes.putredinis	GroupTP	Tx4	0,07746402	0,01772159	34	34	0,00012217	0,00345969
Barnesiella.sp900538555	GroupTP	Tx4	0,00418115	0,00084414	34	34	0,00014386	0,00389674
Alistipes.senegalensis	GroupTP	Tx4	0,02006117	0,00420609	34	33	0,00020891	0,00423035
Parabacteroides.goldsteinii	GroupTP	Tx4	0,02064923	0,00496587	34	34	0,00022408	0,00423035

GCA.900066575.sp900553635	GroupTP	Tx4	0,00752328	0,00156471	34	34	0,00019311	0,00423035
Oscillibacter.sp001916835	GroupTP	Tx4	0,02579172	0,00541897	34	34	0,00021323	0,00423035
CAG.170.sp002404795	GroupTP	Tx4	0,01626193	0,00333935	34	34	0,00017033	0,00423035
CAG.170.sp003516765	GroupTP	Tx4	0,01133413	0,00232892	34	33	0,00017141	0,00423035
PeH17.sp000435055	GroupTP	Tx4	0,00280987	0,00058086	34	34	0,00018192	0,00423035
QAMH01.sp900544245	GroupTP	Tx4	0,01122877	0,0023454	34	30	0,00020137	0,00423035
Zag111.sp003258735	GroupTP	Tx4	0,00491457	0,00103747	34	29	0,00022326	0,00423035
CAG.306.sp000980375	GroupTP	Tx4	0,00717373	0,00150644	34	31	0,00021214	0,00423035
Faecalibacterium.sp900539885	GroupTP	Tx4	0,0248931	0,00603204	34	34	0,00024493	0,0042387
CAG.110.sp900546075	GroupTP	Tx4	0,00776958	0,00165271	34	34	0,00024031	0,0042387
CAG.83.sp003487665	GroupTP	Tx4	0,00907813	0,0019305	34	34	0,00023964	0,0042387
UBA7173.sp900540205	GroupTP	Tx4	0,00371125	0,00079589	34	34	0,00025981	0,0043747
CAG.170.sp000432135	GroupTP	Tx4	0,02130037	0,00462116	34	34	0,00029015	0,00475691
Alistipes.obesi	GroupTP	Tx4	0,01206406	0,00272205	34	34	0,00041853	0,00650967
Butyricimonas.sp002161485	GroupTP	Tx4	0,01556447	0,00395671	34	34	0,0004218	0,00650967
Sutterella.wadsworthensis_A	GroupTP	Tx4	0,02858421	0,00646594	34	34	0,00042841	0,00650967
UMGS882.sp900546385	GroupTP	Tx4	0,00596011	0,00136647	34	32	0,00048431	0,00718387
CAG.170.sp900549635	GroupTP	Tx4	0,01219329	0,00282102	34	34	0,00052566	0,00761596
CAG.95.sp900066375	GroupTP	Tx4	0,01298666	0,0030254	34	34	0,00055928	0,00791893
CAG.170.sp900556635	GroupTP	Tx4	0,00572947	0,00134107	34	34	0,00058339	0,0079686
UMGS1540.sp900552775	GroupTP	Tx4	0,00885502	0,00207463	34	24	0,00058837	0,0079686
CAG.110.sp900544405	GroupTP	Tx4	0,00744117	0,0017505	34	33	0,00061007	0,00808667
Blautia_A.sp900551715	GroupTP	Tx4	0,00477144	0,00125892	34	34	0,0006292	0,00816648
UBA1820.sp002314265	GroupTP	Tx4	0,00979546	0,00233389	34	31	0,00068269	0,00867995
Prevotella.sp900544825	GroupTP	Tx4	0,01807967	0,00439776	34	34	0,00081702	0,01002871
Coprococcus.eutactus_A	GroupTP	Tx4	0,10470381	0,02548177	34	34	0,00082097	0,01002871
Faecalibacterium.prausnitzii_H	GroupTP	Tx4	0,00530261	0,00132474	34	34	0,00102608	0,01219778
Gemmiger.sp900539695	GroupTP	Tx4	0,01887046	0,00522979	34	34	0,00103769	0,01219778
UBA10677.sp900553005	GroupTP	Tx4	0,00388709	0,00097888	34	27	0,00109702	0,01265631
Butyrivibrio_A.sp000431815	GroupTP	Tx4	0,02694486	0,00752438	34	34	0,00111778	0,01266141
Angelakisella.sp900547385	GroupTP	Tx4	0,0185753	0,00521878	34	34	0,00118575	0,01273663

UMGS1071.sp900542375	GroupTP	Tx4	-0,0122134	0,00309834	34	34	0,00116616	0,01273663
Allisonella.histaminiformans	GroupTP	Tx4	0,01156615	0,00293637	34	34	0,00117352	0,01273663
Flavonifractor.plautii	GroupTP	Tx4	-0,0237026	0,00609957	34	34	0,00131203	0,01382598
UMGS1815.sp900550625	GroupTP	Tx4	0,00982762	0,00253358	34	33	0,00133155	0,01382598
Barnesiella.intestinihominis	GroupTP	Tx4	0,03989343	0,01047138	34	34	0,00154068	0,01437322
Blautia_A.sp900066145	GroupTP	Tx4	0,03140379	0,00902942	34	34	0,00147824	0,01437322
Anaerostipes.hadrus	GroupTP	Tx4	-0,0173908	0,00452761	34	34	0,0014423	0,01437322
Coprococcus.sp900548215	GroupTP	Tx4	0,01069142	0,00279314	34	33	0,00148332	0,01437322
Faecalibacterium.prausnitzii_J	GroupTP	Tx4	0,02712823	0,00711714	34	34	0,00153446	0,01437322
CAG.110.sp000434635	GroupTP	Tx4	0,01401031	0,00367305	34	34	0,00152584	0,01437322
SFFH01.sp900542445	GroupTP	Tx4	0,0135721	0,00356391	34	33	0,00154576	0,01437322
UMGS621.sp900543915	GroupTP	Tx4	0,00779083	0,00205864	34	28	0,00162519	0,01488961
UMGS973.sp900547295	GroupTP	Tx4	0,01371176	0,00363259	34	34	0,00165915	0,01498041
CAG.238.sp900542245	GroupTP	Tx4	0,00694251	0,00184304	34	34	0,00168655	0,01501028
UMGS1600.sp900553315	GroupTP	Tx4	0,01073892	0,0028572	34	33	0,00171504	0,01504886
Agathobaculum.sp900291975	GroupTP	Tx4	-0,0037806	0,00101717	34	34	0,00187482	0,01622242
Lachnospira.sp900316325	GroupTP	Tx4	0,02337874	0,00642967	34	34	0,00222319	0,01800545
Massilioclostridium.coli	GroupTP	Tx4	-0,0036507	0,00100221	34	34	0,00219265	0,01800545
Firm.11.sp900548145	GroupTP	Tx4	0,0099055	0,0027098	34	34	0,00213404	0,01800545
Eggerthella.lenta	GroupTP	Tx4	-0,0148506	0,00406502	34	34	0,00214381	0,01800545
Collinsella.bouchesdurhonensis	GroupTP	Tx4	0,00695273	0,00209092	34	33	0,00222539	0,01800545
TF01.11.sp000436755	GroupTP	Tx4	0,01254023	0,00345842	34	34	0,00227097	0,01813865
CAG.83.sp000435555	GroupTP	Tx4	0,04909099	0,01358426	34	34	0,00233021	0,01814651
Collinsella.sp900541235	GroupTP	Tx4	0,00371135	0,00102663	34	31	0,00232392	0,01814651
TF01.11.sp003529475	GroupTP	Tx4	0,02209873	0,00671262	34	34	0,00242949	0,01868611
Blautia.sp001304935	GroupTP	Tx4	-0,0045144	0,00126059	34	34	0,00249645	0,0189669
Alistipes_A.indistinctus	GroupTP	Tx4	0,00965405	0,00270442	34	34	0,00255765	0,01919776
An200.sp003268275	GroupTP	Tx4	-0,0040686	0,00114394	34	34	0,00262906	0,01949883
QALS01.sp003150575	GroupTP	Tx4	0,00776186	0,00219193	34	30	0,00271708	0,01991462
CAG.103.sp900317855	GroupTP	Tx4	0,0346777	0,00984194	34	34	0,00282028	0,02043065
Evtepia.gabavorous	GroupTP	Tx4	0,01376609	0,00393393	34	34	0,0029678	0,02101064
ER4.sp000765235	GroupTP	Tx4	0,02767434	0,00790203	34	34	0,00294992	0,02101064

Faecalibacterium.prausnitzii_I	GroupTP	Tx4	0,01841633	0,00535075	34	34	0,00335093	0,02333899
CAG.495.sp000436375	GroupTP	Tx4	0,03482762	0,01012752	34	32	0,0033716	0,02333899
F23.B02.sp002472405	GroupTP	Tx4	0,0052192	0,00152715	34	33	0,00352668	0,02414423
Butyricimonas.virosa	GroupTP	Tx4	0,01103353	0,00323849	34	34	0,00360652	0,02415982
CAG.110.sp900544945	GroupTP	Tx4	0,00335373	0,00098335	34	34	0,0035799	0,02415982
Prevotella.sp900548745	GroupTP	Tx4	0,00303066	0,00089741	34	34	0,0038411	0,02518955
Clostridium_Q.saccharolyticum_A	GroupTP	Tx4	-0,0060439	0,00178847	34	34	0,00382311	0,02518955
Blautia_A.sp003478765	GroupTP	Tx4	0,00492366	0,00146521	34	34	0,00397885	0,02582106
UBA738.sp003522945	GroupTP	Tx4	0,00769307	0,00230383	34	34	0,00416048	0,02672146
RC9.sp000434935	GroupTP	Tx4	0,0017852	0,0005364	34	34	0,00425904	0,02690384
UBA11774.sp003507655	GroupTP	Tx4	0,01635829	0,00531819	34	34	0,00427525	0,02690384
CAG.83.sp900552475	GroupTP	Tx4	0,00575003	0,00177176	34	34	0,00507007	0,03127838
Parasutterella.sp000980495	GroupTP	Tx4	-0,006568	0,00202384	34	34	0,00507081	0,03127838
CAG.45.sp000438375	GroupTP	Tx4	0,01147108	0,0038413	34	34	0,00538012	0,0328609
Collinsella.sp900541475	GroupTP	Tx4	0,00330908	0,00110979	34	28	0,00544261	0,03291989
Eubacterium_R.sp000433975	GroupTP	Tx4	0,03776709	0,01271561	34	34	0,00560533	0,03357809
Phocaeicola.sp900551445	GroupTP	Tx4	0,00685563	0,00215565	34	33	0,00581327	0,0341667
Muricomes.contorta_B	GroupTP	Tx4	-0,0036583	0,001149	34	34	0,00576915	0,0341667
Flavonifractor.sp000508885	GroupTP	Tx4	-0,0130241	0,00410436	34	34	0,00590015	0,0343532
CAG.83.sp900545495	GroupTP	Tx4	0,0071685	0,00243785	34	34	0,006043	0,03485914
UBA5446.sp900545405	GroupTP	Tx4	0,00313588	0,0009972	34	34	0,00626478	0,03580695
Bacteroides.fragilis	GroupTP	Tx4	-0,0395441	0,01275456	34	34	0,00687422	0,03823783
CAG.110.sp900544705	GroupTP	Tx4	0,00183167	0,00059068	34	34	0,00686604	0,03823783
Collinsella.sp900540945	GroupTP	Tx4	0,00535858	0,00185164	34	30	0,00679576	0,03823783
Erysipelatoclostridium.spiroforme	GroupTP	Tx4	-0,0095936	0,00310088	34	34	0,00696927	0,03842352
Enterocloster.sp005845215	GroupTP	Tx4	-0,0029099	0,00094937	34	34	0,00740105	0,04044611
CAG.81.sp900066055	GroupTP	Tx4	0,00430883	0,00141151	34	34	0,00759673	0,04115446
Blautia_A.sp000285855	GroupTP	Tx4	0,00693835	0,00227763	34	34	0,00769802	0,04134367
Bacteroides.sp002491635	GroupTP	Tx4	-0,0042028	0,0013837	34	34	0,00784269	0,04140673
UMGS1766.sp900554855	GroupTP	Tx4	0,00687167	0,00226121	34	34	0,00781743	0,04140673
Phocaeicola.sp900551065	GroupTP	Tx4	0,0265601	0,00885174	34	33	0,00846973	0,04160752
Prevotella.copri	GroupTP	Tx4	0,17160275	0,05694302	34	34	0,00824247	0,04160752

Dorea.sp000433215	GroupTP	Tx4	0,005501	0,0019468	34	34	0,00806204	0,04160752
Eubacterium_G.sp900548465	GroupTP	Tx4	-0,0035672	0,00118685	34	34	0,0083814	0,04160752
Coprococcus.sp900557435	GroupTP	Tx4	0,00381669	0,00127158	34	31	0,00845242	0,04160752
Coprococcus.sp900548315	GroupTP	Tx4	0,0037874	0,00126016	34	30	0,00838266	0,04160752
Coprococcus_A.catus	GroupTP	Tx4	0,01437188	0,00511642	34	34	0,00840337	0,04160752
UMGS1384.sp900551265	GroupTP	Tx4	-0,0017633	0,00058693	34	34	0,00840353	0,04160752
Holdemanella.sp900547815	GroupTP	Tx4	0,00283918	0,00094643	34	33	0,00848179	0,04160752
Mediterraneibacter.torques	GroupTP	Tx4	-0,0159019	0,00532078	34	34	0,00868292	0,04193377
CAG.83.sp900545585	GroupTP	Tx4	0,02067837	0,00691582	34	34	0,00865798	0,04193377
CAG.95.sp000438155	GroupTP	Tx4	0,00882661	0,00296178	34	34	0,00883748	0,04235191
Prevotella.sp900552515	GroupTP	Tx4	0,00162226	0,00054611	34	34	0,00901579	0,04287663
Enterocloster.asparagiformis	GroupTP	Tx4	-0,0142807	0,00482749	34	34	0,00925111	0,04340049
Massiliomicrobiota.sp002160815	GroupTP	Tx4	-0,0060184	0,00203498	34	34	0,00926527	0,04340049
Eubacterium_G.sp900556905	GroupTP	Tx4	-0,0026704	0,00096577	34	34	0,00936694	0,04354928
CAG.103.sp000432375	GroupTP	Tx4	0,01449477	0,00491818	34	34	0,00946591	0,04368342
CAG.81.sp000435795	GroupTP	Tx4	-0,0157176	0,00535907	34	34	0,00975111	0,04434264
CAG.110.sp000435995	GroupTP	Tx4	0,0205062	0,006987	34	34	0,00971062	0,04434264
UBA1191.sp900066305	GroupTP	Tx4	0,0034375	0,0012562	34	34	0,01005067	0,04537369
Bacteroides.sp900556625	GroupTP	Tx4	0,01555682	0,00536045	34	33	0,01039458	0,04658866
Enterocloster.sp900547035	GroupTP	Tx4	-0,0027171	0,00094312	34	34	0,01086269	0,04833899
UMGS1071.sp900541905	GroupTP	Tx4	0,00521258	0,00181815	34	34	0,01118101	0,04905472
Slackia_A.isoflavoniconvertens	GroupTP	Tx4	0,01196711	0,00417411	34	34	0,0111808	0,04905472
SFEL01.sp004557245	GroupTP	Tx4	0,01744023	0,00609232	34	34	0,01128142	0,0491491
Gemmiger.qucibialis	GroupTP	Tx4	0,03040399	0,01065669	34	34	0,01150827	0,04978927
**12wk vs. BL, random effects: "ID"**								
Alistipes.sp000434235	GroupTP	Tx12	0,0851989	0,01294494	30	30	3,88E-07	0,00023302
Holdemanella.sp003436425	GroupTP	Tx12	0,00863952	0,00143729	30	28	1,78E-06	0,00053304
UMGS1540.sp900552775	GroupTP	Tx12	0,00675228	0,00108567	30	21	1,03E-05	0,00206504
Prevotella.sp000435635	GroupTP	Tx12	0,00496542	0,00097952	30	29	2,30E-05	0,00344282
Alistipes_A.sp900240235	GroupTP	Tx12	0,01231538	0,00273225	30	29	0,00010648	0,00919782
GCA.900066575.sp900553635	GroupTP	Tx12	0,00839224	0,00162717	30	30	0,00010731	0,00919782
CAG.103.sp900317855	GroupTP	Tx12	0,01639368	0,00320945	30	30	0,00010036	0,00919782

CAG.238.sp900551415	GroupTP	Tx12	0,01398252	0,00279767	30	30	0,0001304	0,00977987
CAG.110.sp900544405	GroupTP	Tx12	0,00679502	0,00138487	30	29	0,00025724	0,01714963
Sutterella.wadsworthensis_A	GroupTP	Tx12	0,02931566	0,0064322	30	30	0,00031719	0,01903113
CAG.170.sp900553545	GroupTP	Tx12	0,01245339	0,00310341	30	29	0,00040602	0,02214667
Parabacteroides.goldsteinii	GroupTP	Tx12	0,01709835	0,00413805	30	30	0,00074327	0,02795203
Butyricimonas.sp002161485	GroupTP	Tx12	0,01231496	0,00325354	30	30	0,00074539	0,02795203
CAG.103.sp900543625	GroupTP	Tx12	0,01696461	0,00407099	30	30	0,00072136	0,02795203
UMGS1600.sp900553315	GroupTP	Tx12	0,00933717	0,00221363	30	29	0,00062242	0,02795203
PeH17.sp000435055	GroupTP	Tx12	0,00243112	0,00052416	30	30	0,00072835	0,02795203
Lachnospira.sp900316325	GroupTP	Tx12	0,01988337	0,00476561	30	30	0,00083536	0,0294832
Faecalibacterium.prausnitzii_E	GroupTP	Tx12	0,03831364	0,01050335	30	30	0,00107123	0,03493502
UMGS1071.sp900542375	GroupTP	Tx12	-0,0134668	0,00336061	30	30	0,0011645	0,03493502
Flavonifractor.plautii	GroupTP	Tx12	-0,0168522	0,00396086	30	30	0,00113852	0,03493502
Butyrivibrio_A.sp000431815	GroupTP	Tx12	0,02895466	0,00815548	30	30	0,00138271	0,03899909
CAG.95.sp900066375	GroupTP	Tx12	0,01105655	0,00289201	30	30	0,00149496	0,03899909
CAG.170.sp002404795	GroupTP	Tx12	0,01960378	0,00514084	30	30	0,00145988	0,03899909
Alistipes.shahii	GroupTP	Tx12	0,02420675	0,00623804	30	30	0,00157328	0,03933198
Prevotella.copri	GroupTP	Tx12	0,1871957	0,04698089	30	30	0,00171195	0,03950645
Prevotella.sp900544825	GroupTP	Tx12	0,01608174	0,00420205	30	30	0,00169162	0,03950645
**26wk vs. BL, random effects: "ID"**								
Alistipes.sp000434235	GroupTP	Tx26	0,07468736	0,01220476	33	33	8,74E-07	0,00053557
CAG.238.sp900551415	GroupTP	Tx26	0,01241323	0,00173585	33	33	2,47E-06	0,00075796
Prevotella.sp000435635	GroupTP	Tx26	0,0057874	0,00105819	33	32	5,59E-06	0,00114288
Alistipes.sp900548155	GroupTP	Tx26	0,00516153	0,00097665	33	31	9,49E-06	0,00145379
CAG.83.sp900549395	GroupTP	Tx26	0,00392073	0,00067269	33	33	3,08E-05	0,00377739
Zag111.sp003258735	GroupTP	Tx26	0,00577423	0,00121649	33	29	4,43E-05	0,00453063
Alistipes_A.sp900240235	GroupTP	Tx26	0,01041044	0,00225257	33	32	6,34E-05	0,00504384
ER4.sp900317525	GroupTP	Tx26	0,01975057	0,00371967	33	33	7,04E-05	0,00504384
CAG.170.sp002404795	GroupTP	Tx26	0,01423268	0,00269005	33	33	7,41E-05	0,00504384
Holdemanella.sp003436425	GroupTP	Tx26	0,00690095	0,00155162	33	31	0,00010387	0,00636744
CAG.110.sp900544405	GroupTP	Tx26	0,0074452	0,00147022	33	32	0,00012426	0,00692458
CAG.103.sp900543625	GroupTP	Tx26	0,02068428	0,00417723	33	33	0,00014114	0,00720972

Alistipes.senegalensis	GroupTP	Tx26	0,01394825	0,00286356	33	32	0,00018954	0,00788523
GCA.900066575.sp900553635	GroupTP	Tx26	0,01267032	0,00299657	33	33	0,00019295	0,00788523
Faecalibacterium.sp900539885	GroupTP	Tx26	0,0199098	0,00467133	33	33	0,00017542	0,00788523
Blautia_A.sp900551715	GroupTP	Tx26	0,00416663	0,00087562	33	33	0,00022262	0,00849382
CAG.110.sp900546075	GroupTP	Tx26	0,00412651	0,00086795	33	33	0,0002394	0,00849382
Victivallis.sp002998355	GroupTP	Tx26	0,00801687	0,00170982	33	32	0,00024941	0,00849382
Olsenella_E.sp003609875	GroupTP	Tx26	0,00728517	0,00160567	33	33	0,00034575	0,01115496
UBA738.sp003522945	GroupTP	Tx26	0,00541648	0,00119658	33	33	0,00038721	0,01130283
UMGS1600.sp900553315	GroupTP	Tx26	0,008663	0,00193642	33	32	0,00037927	0,01130283
Alistipes.shahii	GroupTP	Tx26	0,02568602	0,00588237	33	33	0,00053463	0,01489673
Alistipes.putredinis	GroupTP	Tx26	0,06374921	0,01657504	33	33	0,00055925	0,01490517
UMGS1071.sp900542375	GroupTP	Tx26	-0,0138585	0,00323374	33	33	0,00061337	0,0156664
UBA1820.sp002314265	GroupTP	Tx26	0,00710563	0,00172483	33	31	0,0008145	0,01610603
Ruminococcus_C.callidus	GroupTP	Tx26	0,03238963	0,00789211	33	33	0,0008134	0,01610603
CAG.83.sp000435555	GroupTP	Tx26	0,02794928	0,00678326	33	33	0,00079788	0,01610603
ER4.sp003522105	GroupTP	Tx26	0,00337413	0,0008031	33	33	0,0007549	0,01610603
ER4.sp900552015	GroupTP	Tx26	0,00886909	0,00213299	33	32	0,00072229	0,01610603
SFFH01.sp900542395	GroupTP	Tx26	0,02128706	0,00573518	33	33	0,0008084	0,01610603
Sutterella.wadsworthensis_A	GroupTP	Tx26	0,02942104	0,00712815	33	33	0,00077457	0,01610603
An200.sp003268275	GroupTP	Tx26	-0,0041804	0,00101353	33	33	0,00088508	0,01695488
UMGS1815.sp900550625	GroupTP	Tx26	0,00692036	0,0017198	33	32	0,00097565	0,01768958
UMGS1540.sp900552775	GroupTP	Tx26	0,0084072	0,00209517	33	22	0,00098115	0,01768958
CAG.81.sp900066055	GroupTP	Tx26	0,00510568	0,00126446	33	33	0,00103192	0,01807328
Eubacterium_G.sp900556905	GroupTP	Tx26	-0,0035964	0,00091123	33	33	0,00118487	0,018343
CAG.95.sp900066375	GroupTP	Tx26	0,01289705	0,00324488	33	33	0,00109961	0,018343
UMGS1826.sp900555435	GroupTP	Tx26	0,0055718	0,00140845	33	33	0,0011176	0,018343
UMGS1384.sp900551265	GroupTP	Tx26	-0,0018329	0,00046158	33	33	0,00119693	0,018343
UMGS1696.sp900554225	GroupTP	Tx26	0,01470288	0,00373168	33	29	0,00115278	0,018343
Eggerthella.lenta	GroupTP	Tx26	-0,014872	0,00373107	33	33	0,00123519	0,01846758
Prevotella.sp900544825	GroupTP	Tx26	0,01985693	0,00510754	33	33	0,00130905	0,0191059
SFJ001.sp004555865	GroupTP	Tx26	0,00702833	0,00183252	33	31	0,00144145	0,02054909
Massilioclostridium.coli	GroupTP	Tx26	-0,0044356	0,00117182	33	33	0,00169923	0,02314735

CAG.345.sp000433315	GroupTP	Tx26	0,00409836	0,00108115	33	30	0,00166808	0,02314735
Faecalibacterium.prausnitzii_I	GroupTP	Tx26	0,02335629	0,0062932	33	33	0,00190115	0,02533487
CAG.83.sp900552475	GroupTP	Tx26	0,00738502	0,00199753	33	33	0,00199547	0,02602602
Clostridium_Q.saccharolyticum_A	GroupTP	Tx26	-0,0063613	0,00172625	33	33	0,00204127	0,02606869
Angelakisella.sp900547385	GroupTP	Tx26	0,01763673	0,00482665	33	33	0,00211628	0,02647515
Firm.11.sp900548145	GroupTP	Tx26	0,01039726	0,00286368	33	33	0,00221427	0,02714701
CAG.495.sp000436375	GroupTP	Tx26	0,03963238	0,01096345	33	32	0,00228354	0,02744728
Parasutterella.sp000980495	GroupTP	Tx26	-0,007495	0,00226054	33	33	0,00233912	0,02757462
Oscillibacter.sp900544615	GroupTP	Tx26	0,00792987	0,00220335	33	33	0,00244079	0,02823022
CAG.83.sp003487665	GroupTP	Tx26	0,00567675	0,00157531	33	33	0,00255862	0,028517
CAG.103.sp900317855	GroupTP	Tx26	0,02097934	0,00588615	33	33	0,00255072	0,028517
UMGS966.sp900547185	GroupTP	Tx26	-0,0069703	0,00194248	33	33	0,00273493	0,02993775
CAG.170.sp900553545	GroupTP	Tx26	0,01003044	0,00288193	33	32	0,00311831	0,03353547
UMGS973.sp900547295	GroupTP	Tx26	0,00655351	0,00189629	33	33	0,00326365	0,03390874
UMGS1603.sp900553265	GroupTP	Tx26	0,004766	0,00137884	33	32	0,00321565	0,03390874
Phocaeicola.sp900551065	GroupTP	Tx26	0,02043367	0,00605762	33	32	0,00382679	0,03722851
Prevotella.copri	GroupTP	Tx26	0,21581338	0,06337788	33	33	0,00373405	0,03722851
Blautia.sp001304935	GroupTP	Tx26	-0,0041395	0,0012277	33	33	0,00388683	0,03722851
Acutalibacter.sp000435395	GroupTP	Tx26	-0,0079449	0,00232608	33	33	0,00371666	0,03722851
SFFH01.sp900548125	GroupTP	Tx26	0,00813498	0,00241158	33	33	0,00385464	0,03722851
Phocaeicola.sp900551445	GroupTP	Tx26	0,00516582	0,00153834	33	31	0,00398357	0,03756814
Lachnospira.sp900316325	GroupTP	Tx26	0,02421732	0,00724217	33	33	0,00409286	0,03765573
Faecalibacterium.prausnitzii_J	GroupTP	Tx26	0,02507369	0,00748865	33	33	0,00413781	0,03765573
Flavonifractor.plautii	GroupTP	Tx26	-0,0200878	0,00599684	33	33	0,00417714	0,03765573
Butyricimonas.sp002161485	GroupTP	Tx26	0,01449928	0,00473727	33	33	0,00453194	0,04026201
CAG.177.sp003514385	GroupTP	Tx26	0,03753899	0,01149575	33	33	0,00483508	0,04175321
Erysipelatoclostridium.spiroforme	GroupTP	Tx26	-0,0088342	0,00260612	33	33	0,00483602	0,04175321
QALS01.sp003150575	GroupTP	Tx26	0,00580621	0,00178607	33	29	0,00497876	0,04238865
CAG.170.sp900556635	GroupTP	Tx26	0,00304853	0,00095029	33	33	0,00546188	0,04586479
Enterocloster.asparagiformis	GroupTP	Tx26	-0,01563	0,00495412	33	33	0,00612555	0,04748894
Butyrivibrio_A.sp000431815	GroupTP	Tx26	0,03012582	0,00957227	33	33	0,00615025	0,04748894
Lawsonibacter.sp002160305	GroupTP	Tx26	-0,0035354	0,00111392	33	33	0,00610423	0,04748894

Flavonifractor.sp002161085	GroupTP	Tx26	-0,0026917	0,00085485	33	33	0,00627505	0,04748894
CAG.170.sp000432135	GroupTP	Tx26	0,01956825	0,00617578	33	33	0,00591322	0,04748894
CAG.170.sp003516765	GroupTP	Tx26	0,00827358	0,00262972	33	32	0,00626633	0,04748894
CAG.238.sp000435615	GroupTP	Tx26	0,00581581	0,0018201	33	33	0,00574278	0,04748894
Allisonella.histaminiformans	GroupTP	Tx26	0,00741546	0,00233934	33	33	0,00600758	0,04748894

52wk vs. BL, random effects: "ID"

GCA.900066575.sp900553635	GroupTP	Tx52	0,01211921	0,00149734	33	33	4,92E-07	0,00031281
CAG.83.sp900549395	GroupTP	Tx52	0,00610932	0,00081637	33	33	1,65E-06	0,00052313
CAG.103.sp900543625	GroupTP	Tx52	0,02151639	0,00322748	33	33	5,45E-06	0,00115601
Alistipes.sp900548155	GroupTP	Tx52	0,0060305	0,00115293	33	31	1,11E-05	0,00117526
Oscillibacter.sp001916835	GroupTP	Tx52	0,01995064	0,00308515	33	33	9,53E-06	0,00117526
CAG.238.sp900551415	GroupTP	Tx52	0,01447177	0,00226195	33	33	8,87E-06	0,00117526
Alistipes.sp000434235	GroupTP	Tx52	0,08499092	0,01391493	33	33	1,43E-05	0,00125556
CAG.170.sp900549635	GroupTP	Tx52	0,0196749	0,00388359	33	33	1,78E-05	0,00125556
Victivallis.sp002998355	GroupTP	Tx52	0,00932148	0,00183502	33	32	1,71E-05	0,00125556
Prevotella.sp000435635	GroupTP	Tx52	0,00574487	0,00114994	33	32	2,17E-05	0,00125687
CAG.170.sp002404795	GroupTP	Tx52	0,02194786	0,00368334	33	33	2,01E-05	0,00125687
Holdemanella.sp003436425	GroupTP	Tx52	0,00802621	0,00163361	33	31	2,75E-05	0,00145981
Sutterella.wadsworthensis_A	GroupTP	Tx52	0,03225091	0,00566546	33	33	3,36E-05	0,00164221
ER4.sp900552015	GroupTP	Tx52	0,00868954	0,00183934	33	32	4,73E-05	0,00214744
Butyricimonas.sp002161485	GroupTP	Tx52	0,0161034	0,00298333	33	33	5,90E-05	0,00220828
ER4.sp900317525	GroupTP	Tx52	0,02614964	0,00484387	33	33	5,87E-05	0,00220828
UMGS1600.sp900553315	GroupTP	Tx52	0,01076965	0,00197731	33	32	5,24E-05	0,00220828
CAG.170.sp900553545	GroupTP	Tx52	0,01651501	0,00311706	33	32	7,26E-05	0,00256347
CAG.170.sp000432135	GroupTP	Tx52	0,0321236	0,00628234	33	33	0,00010339	0,0034136
UMGS1696.sp900554225	GroupTP	Tx52	0,01542175	0,00348338	33	29	0,00011004	0,0034136
CAG.345.sp000433315	GroupTP	Tx52	0,00688807	0,00135784	33	30	0,00011808	0,0034136
Zag111.sp003258735	GroupTP	Tx52	0,00652826	0,0014789	33	29	0,00011415	0,0034136
CAG.110.sp900546075	GroupTP	Tx52	0,00887022	0,00177222	33	33	0,00012606	0,00348573
SFJ001.sp004555865	GroupTP	Tx52	0,00825332	0,00169551	33	31	0,00016781	0,00444693
Oscillibacter.sp900544615	GroupTP	Tx52	0,00984876	0,00205833	33	33	0,00021326	0,00542523
CAG.170.sp900556635	GroupTP	Tx52	0,005754	0,00122508	33	33	0,00023751	0,00574417

Firm.11.sp900548145	GroupTP	Tx52	0,00922976	0,00222681	33	33	0,00024386	0,00574417
CAG.95.sp900066375	GroupTP	Tx52	0,01131923	0,00243802	33	33	0,00027266	0,0061933
UBA1820.sp002314265	GroupTP	Tx52	0,00858931	0,00192412	33	31	0,00039914	0,00775033
Angelakisella.sp900547385	GroupTP	Tx52	0,0226142	0,00505416	33	33	0,00037962	0,00775033
CAG.83.sp001916855	GroupTP	Tx52	0,00379368	0,0008316	33	33	0,00038033	0,00775033
CAG.103.sp900317855	GroupTP	Tx52	0,02496539	0,00561974	33	33	0,00040214	0,00775033
UBA10677.sp900553005	GroupTP	Tx52	0,00477178	0,00106654	33	27	0,00038385	0,00775033
Holdemanella.biformis	GroupTP	Tx52	0,00955714	0,00215978	33	33	0,00044129	0,00825472
CAG.83.sp000435555	GroupTP	Tx52	0,04626255	0,01055584	33	33	0,0004556	0,00827883
Butyricimonas.virosa	GroupTP	Tx52	0,00928884	0,00212086	33	33	0,00055797	0,00933862
Barnesiella.sp900538555	GroupTP	Tx52	0,00588494	0,00152889	33	33	0,00055454	0,00933862
CAG.170.sp003516765	GroupTP	Tx52	0,01126821	0,00261941	33	32	0,0005508	0,00933862
UMGS1540.sp900552775	GroupTP	Tx52	0,00857467	0,00201421	33	22	0,00058467	0,00953465
Alistipes.putredinis	GroupTP	Tx52	0,06279472	0,01476389	33	33	0,00064109	0,00970788
Alistipes.senegalensis	GroupTP	Tx52	0,01193839	0,00278254	33	32	0,0006161	0,00970788
Blautia_A.sp900551715	GroupTP	Tx52	0,00474844	0,00112292	33	33	0,00063152	0,00970788
Alistipes_A.sp900240235	GroupTP	Tx52	0,0114268	0,00272653	33	31	0,0006767	0,00978134
CAG.83.sp900548615	GroupTP	Tx52	0,00594486	0,00140263	33	33	0,00066762	0,00978134
CAG.238.sp000435615	GroupTP	Tx52	0,00590159	0,00141304	33	33	0,00074987	0,01059817
Parabacteroides.goldsteinii	GroupTP	Tx52	0,02294135	0,00574141	33	33	0,00103072	0,01399662
CAG.110.sp900544405	GroupTP	Tx52	0,00805874	0,00201348	33	32	0,00103434	0,01399662
CAG.83.sp900545495	GroupTP	Tx52	0,00599993	0,00152245	33	33	0,00119545	0,01583976
Alistipes.shahii	GroupTP	Tx52	0,02423872	0,00613978	33	33	0,00125541	0,01603874
UMGS1071.sp900542375	GroupTP	Tx52	-0,0136274	0,00347387	33	33	0,00128262	0,01603874
UMGS882.sp900546385	GroupTP	Tx52	0,00715067	0,0020198	33	32	0,00128613	0,01603874
UMGS966.sp900547185	GroupTP	Tx52	-0,0074409	0,00188318	33	33	0,00132269	0,0161775
Enterocloster.sp005845215	GroupTP	Tx52	-0,0031623	0,00082072	33	33	0,00149558	0,01794693
Eggerthella.lenta	GroupTP	Tx52	-0,0145702	0,00378367	33	33	0,00162365	0,01912302
Lachnospira.sp900316325	GroupTP	Tx52	0,02406651	0,00641731	33	33	0,00174671	0,0198376
Gemmiger.sp900539695	GroupTP	Tx52	0,02077646	0,00605734	33	33	0,00172792	0,0198376
Agathobaculum.sp003481705	GroupTP	Tx52	0,01248331	0,00329068	33	33	0,00179191	0,01999395
Faecalibacterium.sp900539885	GroupTP	Tx52	0,02550082	0,00753707	33	33	0,00195553	0,02050279

Faecalibacterium.prausnitzii_J	GroupTP	Tx52	0,02981385	0,00801441	33	33	0,00190285	0,02050279
Faecalibacterium.prausnitzii_E	GroupTP	Tx52	0,03687045	0,01090428	33	33	0,00196646	0,02050279
CAG.83.sp900552475	GroupTP	Tx52	0,00956595	0,00256952	33	33	0,00187761	0,02050279
CAG.495.sp000436375	GroupTP	Tx52	0,03173165	0,00864222	33	31	0,00202378	0,02076012
CAG.83.sp003487665	GroupTP	Tx52	0,00616754	0,00167553	33	33	0,0021344	0,02121062
Zag111.sp002102825	GroupTP	Tx52	0,00475786	0,00141914	33	33	0,00212132	0,02121062
UMGS973.sp900547295	GroupTP	Tx52	0,00514932	0,00141825	33	33	0,00226442	0,02215648
UMGS621.sp900543915	GroupTP	Tx52	0,00542006	0,00153489	33	28	0,00273865	0,02639067
Blautia.sp001304935	GroupTP	Tx52	-0,0045568	0,00131406	33	33	0,00313685	0,02977668
UBA7182.sp003481535	GroupTP	Tx52	0,01181559	0,00374014	33	33	0,0035186	0,0325638
Ruminococcus_C.callidus	GroupTP	Tx52	0,02953418	0,00865885	33	33	0,00353471	0,0325638
Lawsonibacter.sp900549405	GroupTP	Tx52	0,00730515	0,00231762	33	33	0,00358407	0,0325638
CAG.110.sp900544945	GroupTP	Tx52	0,00285551	0,00084022	33	33	0,00370748	0,03321071
Bacteroides.sp007097645	GroupTP	Tx52	-0,0056987	0,00167514	33	33	0,00414556	0,03611923
Prevotella.sp900544825	GroupTP	Tx52	0,01772796	0,00532279	33	33	0,00422994	0,03611923
Enterocloster.asparagiformis	GroupTP	Tx52	-0,0169601	0,00511008	33	33	0,00431613	0,03611923
Coprococcus.eutactus_A	GroupTP	Tx52	0,06881435	0,02069625	33	33	0,00428213	0,03611923
ER4.sp900550165	GroupTP	Tx52	0,00336123	0,00100333	33	33	0,00429223	0,03611923
Flavonifractor.plautii	GroupTP	Tx52	-0,0205987	0,00621089	33	33	0,00446462	0,03640384
Olsenella_E.sp003609875	GroupTP	Tx52	0,00784424	0,0023748	33	33	0,00443998	0,03640384
CAG.81.sp900066055	GroupTP	Tx52	0,00464688	0,00142663	33	33	0,00513093	0,03839143
TF01.11.sp003529475	GroupTP	Tx52	0,02190015	0,00674305	33	33	0,00499061	0,03839143
Eubacterium_R.sp003526845	GroupTP	Tx52	0,00452553	0,00138968	33	33	0,00508906	0,03839143
CAG.110.sp000435995	GroupTP	Tx52	0,02190989	0,00670856	33	33	0,0048256	0,03839143
CAG.170.sp000436735	GroupTP	Tx52	0,01069634	0,0032913	33	33	0,00505174	0,03839143
Holdemanella.sp002299315	GroupTP	Tx52	0,0155664	0,00475835	33	33	0,00491203	0,03839143
CAG.1000.sp000434555	GroupTP	Tx52	0,00519712	0,00158886	33	30	0,0048599	0,03839143
Phocaeicola.sp900066455	GroupTP	Tx52	0,00721862	0,00222684	33	33	0,0052378	0,03873535
UMGS1815.sp900550625	GroupTP	Tx52	0,0065803	0,00204992	33	32	0,00543555	0,03973577
Faecalibacterium.prausnitzii_H	GroupTP	Tx52	0,00408904	0,00128453	33	33	0,00613403	0,04433231
Paraprevotella.xylaniphila	GroupTP	Tx52	0,00656725	0,00223718	33	33	0,00622346	0,04447327
Phocaeicola.sp900551445	GroupTP	Tx52	0,00683026	0,00218105	33	31	0,00640045	0,04496509

CAG.611.sp000434175	GroupTP	Tx52	0,00560736	0,00178342	33	28	0,00643368	0,04496509
CAG.110.sp000434635	GroupTP	Tx52	0,0156127	0,00500973	33	33	0,00656675	0,04539624
UMGS1670.sp900553995	GroupTP	Tx52	0,00551299	0,00178091	33	33	0,0069473	0,04651035
CAG.238.sp900542245	GroupTP	Tx52	0,00610198	0,00196532	33	33	0,00680519	0,04651035
SFFH01.sp900548125	GroupTP	Tx52	0,00603463	0,00194679	33	33	0,00689988	0,04651035
Coprococcus.sp900548215	GroupTP	Tx52	0,00808254	0,00261986	33	31	0,00707248	0,04654905
Collinsella.sp900541235	GroupTP	Tx52	0,00462073	0,00160281	33	30	0,00709946	0,04654905
UBA7182.sp003480725	GroupTP	Tx52	0,01087426	0,00350519	33	33	0,00725958	0,04711322
Bacteroides.xylanisolvens	GroupTP	Tx52	-0,0414794	0,0133414	33	33	0,00753354	0,04785759
Bacteroides.ndongoniae	GroupTP	Tx52	0,00305608	0,00099924	33	33	0,00760003	0,04785759
QALS01.sp003150575	GroupTP	Tx52	0,00615701	0,00201375	33	28	0,00748596	0,04785759
Acutalibacter.sp000435395	GroupTP	Tx52	-0,0065067	0,00212739	33	33	0,00781517	0,04872991
CAG.170.sp900545925	GroupTP	Tx52	0,01828267	0,00644247	33	33	0,00794051	0,0490307

Supplementary table 4. Differential abundance testing on functional pathways with Maaslin2

FMT group vs. Placebo group

feature	metadata	value	coef	stderr	N	N.not.0	pval	qval	High *Prevotella copri* contribution
**Baseline n=0**									
**4wk n=7, 7 up**									
PWY-5154 L-arginine biosynthesis III (via N-acetyl L-citrulline)	GroupShort	T	0,6828563	0,1286947	29	29	1,34E-05	0,0045549	
DARABCATK12-PWY D-arabinose degradation I	GroupShort	T	1,9352881 4	0,4268933 8	29	12	0,0001066 8	0,0181363 7	
PWY4FS-7 phosphatidylglycerol biosynthesis I (plastidic)	GroupShort	T	0,9666275 6	0,2446956 8	29	29	0,0005046 5	0,0428952 4	
PWY4FS-8 phosphatidylglycerol biosynthesis II (non plastidic)	GroupShort	T	0,9666275 6	0,2446956 8	29	29	0,0005046 5	0,0428952 4	
PHOSLIPSYN-PWY superpathway of phospholipid biosynthesis I (bacteria)	GroupShort	T	0,7957017 3	0,2061844 6	29	29	0,0006416 1	0,0436296 4	
PWY-7942 5-oxo-L-proline metabolism	GroupShort	T	1,5472582 5	0,4161015 4	29	15	0,0009275 1	0,0525587 6	
PWY0-1241 ADP-L-glycero-beta-D-manno-heptose biosynthesis	GroupShort	T	1,0473392 6	0,2994595 6	29	26	0,0016443 1	0,0798665 8	
**12wk n=4, 2 up, 2 down**									
ARGININE-SYN4-PWY L-ornithine biosynthesis II	GroupShort	T	-1,0567031	0,2723026	26	26	0,0007120 5	0,0866330 5	
PWY-7560 II methylerythritol phosphate pathway II	GroupShort	T	0,5743388 9	0,1372464 2	26	26	0,0003300 5	0,0866330 5	x

PWY-7977 L-methionine biosynthesis IV	GroupShort	T	0,31754004	0,08019796	26	26	0,00058372	0,08663305	x
PWY0-845 superpathway of pyridoxal 5-phosphate biosynthesis and salvage	GroupShort	T	-1,3176091	0,35419424	26	26	0,0010656	0,09723579	
**26wk n=17, 10 up, 7 down**									
COBALSYN-PWY superpathway of adenosylcobalamin salvage (from cobinamide I)	GroupShort	T	-0,9539127	0,24662353	28	28	0,0006592	0,05444605	
PWY-5103 L-isoleucine biosynthesis III	GroupShort	T	0,36561012	0,09485584	28	28	0,00068271	0,05444605	x
PWY-7282 4-amino-2-methyl-5-diphosphomethylpyrimidine biosynthesis II	GroupShort	T	-1,1736019	0,30260757	28	28	0,00064164	0,05444605	
PWY0-845 superpathway of pyridoxal 5'-phosphate biosynthesis and salvage	GroupShort	T	-1,6583403	0,42902783	28	28	0,00066357	0,05444605	
PWY-5154 L-arginine biosynthesis III (via N-acetyl-L-citrulline)	GroupShort	T	0,58246609	0,15928863	28	28	0,00113693	0,0591615	
PWY-7560 methylerythritol phosphate pathway II	GroupShort	T	0,68942385	0,19406155	28	28	0,00148367	0,0591615	
PYRIDOXSYN-PWY pyridoxal 5'-phosphate biosynthesis I	GroupShort	T	-1,6810967	0,46242554	28	28	0,0012007	0,0591615	
ARGININE-SYN4-PWY L-ornithine biosynthesis II	GroupShort	T	-1,2347809	0,36928851	28	28	0,00251743	0,08705325	
BRANCHED-CHAIN-AA-SYN-PWY superpathway of branched chain amino acid biosynthesis	GroupShort	T	0,27570088	0,08325568	28	28	0,00272894	0,08705325	x
ILEUSYN-PWY L-isoleucine biosynthesis I (from threonine)	GroupShort	T	0,27826131	0,0877411	28	28	0,00386714	0,08906725	x
PWY-1042 glycolysis IV	GroupShort	T	0,25183828	0,0804364	28	28	0,00427339	0,08906725	x
PWY-6897 thiamine diphosphate salvage II	GroupShort	T	0,33226699	0,10674021	28	28	0,00446732	0,08906725	x
PWY-6906 chitin derivatives degradation	GroupShort	T	-1,4363572	0,45980168	28	20	0,00434805	0,08906725	

PWY-7221 guanosine ribonucleotides de novo biosynthesis	GroupShort	T	0,28364141	0,0894206	28	28	0,0038614	0,08906725	x
PWY-7977 L-methionine biosynthesis IV	GroupShort	T	0,28833724	0,09015819	28	28	0,00361952	0,08906725	
PWY-5121 superpathway of geranylgeranyl diphosphate biosynthesis II (via MEP)	GroupShort	T	-0,8542669	0,28097149	28	28	0,00533436	0,09453674	
PWY-7942 5-oxo-L-proline metabolism	GroupShort	T	1,23157535	0,40404475	28	12	0,00523493	0,09453674	

Supplementary table 5. Differential abundance testing on functional pathways with Maaslin2

FMT group follow-up time points vs. baseline

Feature	metadata	value	coef	stderr	N	N.not.0	pval	qval	High *Prevotella copri* contribution
**4wk vs. BL, n=7, up=6, down=1**									
DARABCATK12-PWY: D-arabinose degradation I	GroupTP2	Tx4	1.91276778	0.44228103	34	15	0.00052296	0.08574115	
PWY-5130: 2-oxobutanoate degradation I	GroupTP2	Tx4	1.31575277	0.34722165	34	26	0.00160839	0.08574115	
PWY-6608: guanosine nucleotides degradation III	GroupTP2	Tx4	-0.679806	0.18091218	34	34	0.00171974	0.08574115	
PWY-7234: inosine-5'-phosphate biosynthesis III	GroupTP2	Tx4	1.09606149	0.28300122	34	33	0.00134836	0.08574115	
PWY-7761: NAD salvage pathway II (PNC IV cycle)	GroupTP2	Tx4	1.1463418	0.29718407	34	33	0.00139357	0.08574115	
PWY-7942: 5-oxo-L-proline metabolism	GroupTP2	Tx4	1.40794562	0.3346609	34	19	0.00066852	0.08574115	
PWY0-1479: tRNA processing	GroupTP2	Tx4	1.27059506	0.33491378	34	32	0.00159345	0.08574115	
**12wk vs. BL, n=0**									
**26wk vs. BL, n=38, up=12, down=26**									
PWY0-162: superpathway of pyrimidine ribonucleotides de novo biosynthesis	GroupTP2	Tx26	-0.8606686	0.16990135	33	33	0.00013769	0.04791652	
ARG+POLYAMINE-SYN: superpathway of arginine and polyamine biosynthesis	GroupTP2	Tx26	-1.1953758	0.31020409	33	28	0.00150777	0.06558784	

P105-PWY: TCA cycle IV (2-oxoglutarate decarboxylase)	GroupTP2	Tx26	-2.9378389	0.7211963	33	14	0.00100664	0.06558784	
PENTOSE-P-PWY: pentose phosphate pathway	GroupTP2	Tx26	-0.6619899	0.16693723	33	33	0.0013689	0.06558784	
POLYAMSYN-PWY: superpathway of polyamine biosynthesis I	GroupTP2	Tx26	-1.1477475	0.29122939	33	29	0.00126803	0.06558784	
PWY-5103: L-isoleucine biosynthesis III	GroupTP2	Tx26	0.28950711	0.07070573	33	33	0.00091598	0.06558784	x
PWY-5497: purine nucleobases degradation II (anaerobic)	GroupTP2	Tx26	-0.9910674	0.2374407	33	20	0.00081912	0.06558784	
ARGININE-SYN4-PWY: L-ornithine biosynthesis II	GroupTP2	Tx26	-0.8652665	0.22823405	33	32	0.00201185	0.07001236	
PWY-5030: L-histidine degradation III	GroupTP2	Tx26	-0.6656359	0.17562123	33	32	0.0018254	0.07001236	
BRANCHED-CHAIN-AA-SYN-PWY: superpathway of branched chain amino acid biosynthesis	GroupTP2	Tx26	0.2200237	0.06104835	33	33	0.00254727	0.07387092	x
PWY-6902: chitin degradation II (Vibrio)	GroupTP2	Tx26	-1.160205	0.32081812	33	32	0.00238398	0.07387092	
CITRULBIO-PWY: L-citrulline biosynthesis	GroupTP2	Tx26	-0.6096084	0.16978542	33	33	0.00294824	0.07557169	
ILEUSYN-PWY: L-isoleucine biosynthesis I (from threonine)	GroupTP2	Tx26	0.21605206	0.06138457	33	33	0.00304024	0.07557169	x
PWY-5130: 2-oxobutanoate degradation I	GroupTP2	Tx26	0.82732127	0.24068507	33	25	0.00363028	0.0842225	
BIOTIN-BIOSYNTHESIS-PWY: biotin biosynthesis I	GroupTP2	Tx26	-1.1880379	0.34375574	33	29	0.00455604	0.0859695	

COBALSYN-PWY: superpathway of adenosylcobalamin salvage from cobinamide I	GroupTP2	Tx26	-0.5114759	0.15447099	33	33	0.00477031	0.0859695	
PHOSLIPSYN-PWY: superpathway of phospholipid biosynthesis I (bacteria)	GroupTP2	Tx26	0.48777953	0.14374682	33	33	0.0042558	0.0859695	
PWY-6749: CMP-legionaminate biosynthesis I	GroupTP2	Tx26	-1.6422404	0.50078942	33	11	0.00494078	0.0859695	
PWY1ZNC-1: assimilatory sulfate reduction IV	GroupTP2	Tx26	-1.6763395	0.50485448	33	21	0.00442552	0.0859695	
PWY-6897: thiamine diphosphate salvage II	GroupTP2	Tx26	0.26678596	0.08392625	33	33	0.00605423	0.09682269	x
PYRIDOXSYN-PWY: pyridoxal 5'-phosphate biosynthesis I	GroupTP2	Tx26	-1.0196051	0.31630316	33	31	0.00612097	0.09682269	
PWY-1042: glycolysis IV	GroupTP2	Tx26	0.15801715	0.05119561	33	33	0.00733826	0.09821981	x
PWY-6519: 8-amino-7-oxononanoate biosynthesis I	GroupTP2	Tx26	-1.1530346	0.36023235	33	29	0.00718795	0.09821981	
PWY-6572: chondroitin sulfate degradation I (bacterial)	GroupTP2	Tx26	-1.022652	0.3265317	33	12	0.00670727	0.09821981	
PWY-6731: starch degradation III	GroupTP2	Tx26	-1.0960391	0.35236886	33	32	0.00696917	0.09821981	
FASYN-ELONG-PWY: fatty acid elongation -- saturated	GroupTP2	Tx26	-1.1027617	0.37189876	33	29	0.01092658	0.09996913	
HISDEG-PWY: L-histidine degradation I	GroupTP2	Tx26	-0.645217	0.21442612	33	32	0.00911458	0.09996913	
PWY-5022: 4-aminobutanoate degradation V	GroupTP2	Tx26	-1.1724757	0.40054913	33	14	0.01025205	0.09996913	

PWY-5154: L-arginine biosynthesis III (via N-acetyl-L-citrulline)	GroupTP2	Tx26	0.34164002	0.11540697	33	33	0.01052942	0.09996913
PWY-5989: stearate biosynthesis II (bacteria and plants)	GroupTP2	Tx26	-1.1278989	0.38181726	33	29	0.01105936	0.09996913
PWY-6282: palmitoleate biosynthesis I (from (5Z)-dodec-5-enoate)	GroupTP2	Tx26	-1.1467833	0.38761613	33	29	0.01097624	0.09996913
PWY-7199: pyrimidine deoxyribonucleosides salvage	GroupTP2	Tx26	0.16511571	0.05722391	33	33	0.01120344	0.09996913
PWY-7664: oleate biosynthesis IV (anaerobic)	GroupTP2	Tx26	-1.123047	0.37888528	33	29	0.01092289	0.09996913
PWY-7761: NAD salvage pathway II (PNC IV cycle)	GroupTP2	Tx26	0.95834549	0.33253486	33	32	0.01098594	0.09996913
PWY0-862: (5Z)-dodecenoate biosynthesis I	GroupTP2	Tx26	-1.1448128	0.38647631	33	29	0.01093378	0.09996913
PWY4FS-7: phosphatidylglycerol biosynthesis I (plastidic)	GroupTP2	Tx26	0.48029688	0.1654093	33	33	0.01073702	0.09996913
PWY4FS-8: phosphatidylglycerol biosynthesis II (non-plastidic)	GroupTP2	Tx26	0.48016847	0.16543362	33	33	0.01076339	0.09996913
SER-GLYSYN-PWY: superpathway of L-serine and glycine biosynthesis I	GroupTP2	Tx26	-0.3640924	0.12595728	33	33	0.01084013	0.09996913

52wk vs. BL, n=1, up=1, down=0

PWY-5130: 2-oxobutanoate degradation I	GroupTP2	Tx52	1.23247243	0.26096362	33	26	0.00026477	0.0934624

Supplementary table 6. Differential abundance testing on functional pathways with Maaslin2

Placebo group follow-up time points vs. baseline

feature	metadata	value	coef	stderr	N	N.not.0	pval	qval
**26wk vs. BL, n=2, up=0, down=2**								
P161-PWY: acetylene degradation (anaerobic)	GroupTP2	Px26	-1,5850729	0,29650385	25	16	0,00020799	0,07113418
PWY4LZ-257: superpathway of fermentation (Chlamydomonas reinhardtii)	GroupTP2	Px26	-1,6833291	0,34248411	25	14	0,00046983	0,08034139

Supplementary table 7. Metabolic pathway associations with clinical data with MaAsLin2

MaAsLin2 (random effects “ID”)

feature	metadata	value	coef	stderr	N	N.not.0	pval	qval
**FMT Follow-up samples**								
**delta IBS-SSS**								
No associations								
**delta BAI**								
PWY-7111: pyruvate fermentation to isobutanol (engineered)	DeltaBAI	DeltaBAI	0.071	0.01	62	62	1.85355953990964e-05	0.00627
PWY-7328: superpathway of UDP-glucose-derived O-antigen building blocks biosynthesis	DeltaBAI	DeltaBAI	0.075	0.02	62	62	0.00045	0.07682
OANTIGEN-PWY: O-antigen building blocks biosynthesis (E. coli)	DeltaBAI	DeltaBAI	0.065	0.02	62	62	0.00106	0.09187
PWY-6969: TCA cycle V (2-oxoglutarate synthase)	DeltaBAI	DeltaBAI	0.096	0.03	62	62	0.00109	0.09187
**delta BDI**								
PWY-7328: superpathway of UDP-glucose-derived O-antigen building blocks biosynthesis	DeltaBDI.II	DeltaBDI.II	0.058	0.01	62	62	0.00012	0.03899
**delta IBS-QOL**								
PWY-7312: dTDP-β-D-fucofuranose biosynthesis	deltaIBSQoL	deltaIBSQoL	0.024	0	62	7	4.54328438011422e-05	0.01536
**delta 15D**								
No associations								
**Placebo Follow-up samples**								
**delta IBS-SSS**								
No associations								
**delta BAI**								

No associations
**delta BDI**
No associations
**delta IBS-QOL**
No associations
**delta 15D**
No associations
