# Supplementary material for: Fecal microbiota transplantation influences microbiota without connection to symptom relief in irritable bowel syndrome patients
Source: NPJ Biofilms Microbiomes. 2024 Aug 28;10:73. doi: 10.1038/s41522-024-00549-x (PMC11349920; doi:10.1038/s41522-024-00549-x)
Supplement: Supplementary file 2 — Report Summary [file 41522_2024_549_MOESM2_ESM.pdf]

Corresponding author(s): Anna Hartikainen

Last updated by author(s): Jun 5, 2024

Reporting Summary

Nature Portfolio wishes to improve the reproducibility of the work that we publish. This form provides structure for consistency and transparency in reporting. For further information on Nature Portfolio policies, see our Editorial Policies and the Editorial Policy Checklist.

Statistics

For all statistical analyses, confirm that the following items are present in the figure legend, table legend, main text, or Methods section.

n/a	Confirmed	
☐	☒	The exact sample size (*n*) for each experimental group/condition, given as a discrete number and unit of measurement
☐	☒	A statement on whether measurements were taken from distinct samples or whether the same sample was measured repeatedly
☐	☒	The statistical test(s) used AND whether they are one- or two-sided<br>*Only common tests should be described solely by name; describe more complex techniques in the Methods section.*
☐	☒	A description of all covariates tested
☐	☒	A description of any assumptions or corrections, such as tests of normality and adjustment for multiple comparisons
☐	☒	A full description of the statistical parameters including central tendency (e.g. means) or other basic estimates (e.g. regression coefficient) AND variation (e.g. standard deviation) or associated estimates of uncertainty (e.g. confidence intervals)
☐	☒	For null hypothesis testing, the test statistic (e.g. *F*, *t*, *r*) with confidence intervals, effect sizes, degrees of freedom and *P* value noted<br>*Give P values as exact values whenever suitable.*
☒	☐	For Bayesian analysis, information on the choice of priors and Markov chain Monte Carlo settings
☒	☐	For hierarchical and complex designs, identification of the appropriate level for tests and full reporting of outcomes
☒	☐	Estimates of effect sizes (e.g. Cohen's *d*, Pearson's *r*), indicating how they were calculated

Our web collection on statistics for biologists contains articles on many of the points above.

Software and code

Policy information about availability of computer code

Data collection	16S rRNA gene sequencing (MiSeq, Illumina)<br>Shotgun metagenomics (NextSeq 550, Illumina)
Data analysis	R software (version 4.3.0)<br>mare<br>USEARCH<br>RDP database<br>TRIMMOMATIC (version 0.39)<br>Bowtie2 (version 2.3.5.1)<br>samtools (version 1.16.1)<br>human chromosomal database (GRCh38)<br>Kraken2 (version 2.1.0)<br>Bracken (version 2.7.0)<br>HumGut database<br>HUMAnN3 (version 3.0.1)<br>ggplot2<br>miaViz<br>vegan<br>scater<br>Wilcoxon signed rank test<br>MaAsLin2

StrainPhlAn (4.0.6)
MetaPhlAn (version 4.0.2)
MEGA X (version 10.2.6)
iTol (version 6)

For manuscripts utilizing custom algorithms or software that are central to the research but not yet described in published literature, software must be made available to editors and reviewers. We strongly encourage code deposition in a community repository (e.g. GitHub). See the Nature Portfolio guidelines for submitting code & software for further information.

Data

Policy information about availability of data

All manuscripts must include a data availability statement. This statement should provide the following information, where applicable:

- Accession codes, unique identifiers, or web links for publicly available datasets
- A description of any restrictions on data availability
- For clinical datasets or third party data, please ensure that the statement adheres to our policy

Both 16S rRNA gene amplicon and shotgun metagenomics data are publicly available at ENA under accession number PRJEB65418. Clinical samples used for microbiota analysis are not available for other researchers due to restrictions on distribution of clinical material. The code used for the bioinformatic analysis and statistics is available upon request to the corresponding author.

Research involving human participants, their data, or biological material

Policy information about studies with human participants or human data. See also policy information about sex, gender (identity/presentation), and sexual orientation and race, ethnicity and racism.

Reporting on sex and gender	We did not study the sex and gender differences in this study. In the original clinical trial sex of the participants was equally distributed in study groups (Lahtinen et al. info).
Reporting on race, ethnicity, or other socially relevant groupings	All participants are same ethnicity and purpose of the study was to compare treatment and placebo groups and changes in relation to symptoms. Study details can be found in Lahtinen et al. (Aliment Pharmacol Ther. 2020 Jun;51(12):1321-1331. doi: 10.1111/apt.15740.) and ClinicalTrials.gov (NCT03561519).
Population characteristics	Study details can be found in Lahtinen et al. (Aliment Pharmacol Ther. 2020 Jun;51(12):1321-1331. doi: 10.1111/apt.15740.) and ClinicalTrials.gov (NCT03561519).
Recruitment	Study details can be found in Lahtinen et al. (Aliment Pharmacol Ther. 2020 Jun;51(12):1321-1331. doi: 10.1111/apt.15740.) and ClinicalTrials.gov (NCT03561519).
Ethics oversight	All patients received trial info and they gave written informed consent to participate. The clinical trial was approved by the ethical committee of Helsinki University Hospital (registration number 40/13/03/01/2015). The samples from the non-IBS controls were collected with the approval HUS 29/13/03/01/2014. Study details can be found in Lahtinen et al. (Aliment Pharmacol Ther. 2020 Jun;51(12):1321-1331. doi: 10.1111/apt.15740.) and ClinicalTrials.gov (NCT03561519).

Note that full information on the approval of the study protocol must also be provided in the manuscript.

Field-specific reporting

Please select the one below that is the best fit for your research. If you are not sure, read the appropriate sections before making your selection.

☒ Life sciences ☐ Behavioural & social sciences ☐ Ecological, evolutionary & environmental sciences

For a reference copy of the document with all sections, see nature.com/documents/nr-reporting-summary-flat.pdf

Life sciences study design

All studies must disclose on these points even when the disclosure is negative.

Sample size	Sample size in the original clinical trial was calculated based on the assumption of a 40% placebo effect and clinically significant 40% treatment effect over placebo. Sample size of 26 patients in each group was based on a two group x2 test with a 0.05 two-sided significance level with 80% power to detect difference. Study details can be found in Lahtinen et al. (Aliment Pharmacol Ther. 2020 Jun;51(12):1321-1331. doi: 10.1111/apt.15740.) and ClinicalTrials.gov (NCT03561519).
Data exclusions	One sample with less than 10k reads was excluded.
Replication	The analyzed samples are from clinical trial and thereby replication of samples does not apply.
Randomization	Patients participating in the original clinical trial were randomised in 1:1 ratio into FMT and placebo groups. Randomisation was done in block of six by a study nurse who was not involved in the patients' treatment. Study details can be found in Lahtinen et al. (Aliment Pharmacol Ther. 2020 Jun;51(12):1321-1331. doi: 10.1111/apt.15740.) and ClinicalTrials.gov (NCT03561519).

Blinding	Original clinical trial was double-blinded. Study details can be found in Lahtinen et al. (Aliment Pharmacol Ther. 2020 Jun;51(12):1321-1331. doi: 10.1111/apt.15740.) and ClinicalTrials.gov (NCT03561519).

Reporting for specific materials, systems and methods

We require information from authors about some types of materials, experimental systems and methods used in many studies. Here, indicate whether each material, system or method listed is relevant to your study. If you are not sure if a list item applies to your research, read the appropriate section before selecting a response.

Materials & experimental systems

n/a	Involved in the study
☒	☐ Antibodies
☒	☐ Eukaryotic cell lines
☒	☐ Palaeontology and archaeology
☒	☐ Animals and other organisms
☐	☒ Clinical data
☒	☐ Dual use research of concern
☒	☐ Plants

Methods

n/a	Involved in the study
☒	☐ ChIP-seq
☒	☐ Flow cytometry
☒	☐ MRI-based neuroimaging

Clinical data

Policy information about clinical studies

All manuscripts should comply with the ICMJE guidelines for publication of clinical research and a completed CONSORT checklist must be included with all submissions.

Clinical trial registration	ClinicalTrials.gov registration number of the trial is NCT03561519.
Study protocol	Protocol can be found at ClinicalTrials.gov (NCT03561519).
Data collection	We used clinical data that was previously collected Lahtinen et al. (Aliment Pharmacol Ther. 2020 Jun;51(12):1321-1331. doi: 10.1111/apt.15740.)
Outcomes	We correlated microbial findings with previously recorded clinical outcomes (Lahtinen et al. Aliment Pharmacol Ther. 2020 Jun;51(12):1321-1331. doi: 10.1111/apt.15740.)

Plants

Seed stocks	N/A
Novel plant genotypes	N/A
Authentication	N/A
